# Measurement of the mass dependence of the transverse momentum of lepton pairs in Drell–Yan production in proton–proton collisions at $\sqrt{s} = 13\,\text {Te\hspace{-.08em}V}$

**DOI:** 10.1140/epjc/s10052-023-11631-7

**Published:** 2023-07-17

**Authors:** A. Tumasyan, W. Adam, J. W. Andrejkovic, T. Bergauer, S. Chatterjee, M. Dragicevic, A. Escalante Del Valle, R. Frühwirth, M. Jeitler, N. Krammer, L. Lechner, D. Liko, I. Mikulec, P. Paulitsch, F. M. Pitters, J. Schieck, R. Schöfbeck, M. Spanring, S. Templ, W. Waltenberger, C. -E. Wulz, M. R. Darwish, E. A. De Wolf, T. Janssen, T. Kello, A. Lelek, H. Rejeb Sfar, P. Van Mechelen, S. Van Putte, N. Van Remortel, F. Blekman, E. S. Bols, J. D’Hondt, M. Delcourt, H. El Faham, S. Lowette, S. Moortgat, A. Morton, D. Müller, A. R. Sahasransu, S. Tavernier, W. Van Doninck, P. Van Mulders, D. Beghin, B. Bilin, B. Clerbaux, G. De Lentdecker, L. Favart, A. Grebenyuk, A. K. Kalsi, K. Lee, M. Mahdavikhorrami, I. Makarenko, L. Moureaux, L. Pétré, A. Popov, N. Postiau, E. Starling, L. Thomas, M. Vanden Bemden, C. Vander Velde, P. Vanlaer, D. Vannerom, L. Wezenbeek, T. Cornelis, D. Dobur, J. Knolle, L. Lambrecht, G. Mestdach, M. Niedziela, C. Roskas, A. Samalan, K. Skovpen, M. Tytgat, B. Vermassen, M. Vit, A. Bethani, G. Bruno, F. Bury, C. Caputo, P. David, C. Delaere, I. S. Donertas, A. Giammanco, K. Jaffel, Sa. Jain, V. Lemaitre, K. Mondal, J. Prisciandaro, A. Taliercio, M. Teklishyn, T. T. Tran, P. Vischia, S. Wertz, G. A. Alves, C. Hensel, A. Moraes, W. L. Aldá Júnior, M. Alves Gallo Pereira, M. Barroso Ferreira Filho, H. Brandao Malbouisson, W. Carvalho, J. Chinellato, E. M. Da Costa, G. G. Da Silveira, D. De Jesus Damiao, S. Fonseca De Souza, D. Matos Figueiredo, C. Mora Herrera, K. Mota Amarilo, L. Mundim, H. Nogima, P. Rebello Teles, A. Santoro, S. M. Silva Do Amaral, A. Sznajder, M. Thiel, F. Torres Da Silva De Araujo, A. Vilela Pereira, C. A. Bernardes, L. Calligaris, T. R. Fernandez Perez Tomei, E. M. Gregores, D. S. Lemos, P. G. Mercadante, S. F. Novaes, Sandra S. Padula, A. Aleksandrov, G. Antchev, R. Hadjiiska, P. Iaydjiev, M. Misheva, M. Rodozov, M. Shopova, G. Sultanov, A. Dimitrov, T. Ivanov, L. Litov, B. Pavlov, P. Petkov, A. Petrov, T. Cheng, Q. Guo, T. Javaid, M. Mittal, H. Wang, L. Yuan, M. Ahmad, G. Bauer, C. Dozen, Z. Hu, J. Martins, Y. Wang, K. Yi, E. Chapon, G. M. Chen, H. S. Chen, M. Chen, F. Iemmi, A. Kapoor, D. Leggat, H. Liao, Z.-A. Liu, V. Milosevic, F. Monti, R. Sharma, J. Tao, J. Thomas-Wilsker, J. Wang, H. Zhang, S. Zhang, J. Zhao, A. Agapitos, Y. An, Y. Ban, C. Chen, A. Levin, Q. Li, X. Lyu, Y. Mao, S. J. Qian, D. Wang, Q. Wang, J. Xiao, M. Lu, Z. You, X. Gao, H. Okawa, Z. Lin, M. Xiao, C. Avila, A. Cabrera, C. Florez, J. Fraga, J. Mejia Guisao, F. Ramirez, J. D. Ruiz Alvarez, C. A. Salazar González, D. Giljanovic, N. Godinovic, D. Lelas, I. Puljak, Z. Antunovic, M. Kovac, T. Sculac, V. Brigljevic, D. Ferencek, D. Majumder, M. Roguljic, A. Starodumov, T. Susa, A. Attikis, K. Christoforou, E. Erodotou, A. Ioannou, G. Kole, M. Kolosova, S. Konstantinou, J. Mousa, C. Nicolaou, F. Ptochos, P. A. Razis, H. Rykaczewski, H. Saka, M. Finger, M. Finger, A. Kveton, E. Ayala, E. Carrera Jarrin, A. A. Abdelalim, S. Elgammal, A. Lotfy, M. A. Mahmoud, S. Bhowmik, R. K. Dewanjee, K. Ehataht, M. Kadastik, S. Nandan, C. Nielsen, J. Pata, M. Raidal, L. Tani, C. Veelken, P. Eerola, L. Forthomme, H. Kirschenmann, K. Osterberg, M. Voutilainen, S. Bharthuar, E. Brücken, F. Garcia, J. Havukainen, M. S. Kim, R. Kinnunen, T. Lampén, K. Lassila-Perini, S. Lehti, T. Lindén, M. Lotti, L. Martikainen, M. Myllymäki, J. Ott, H. Siikonen, E. Tuominen, J. Tuominiemi, P. Luukka, H. Petrow, T. Tuuva, C. Amendola, M. Besancon, F. Couderc, M. Dejardin, D. Denegri, J. L. Faure, F. Ferri, S. Ganjour, A. Givernaud, P. Gras, G. Hamel de Monchenault, P. Jarry, B. Lenzi, E. Locci, J. Malcles, J. Rander, A. Rosowsky, M. Ö. Sahin, A. Savoy-Navarro, M. Titov, G. B. Yu, S. Ahuja, F. Beaudette, M. Bonanomi, A. Buchot Perraguin, P. Busson, A. Cappati, C. Charlot, O. Davignon, B. Diab, G. Falmagne, S. Ghosh, R. Granier de Cassagnac, A. Hakimi, I. Kucher, J. Motta, M. Nguyen, C. Ochando, P. Paganini, J. Rembser, R. Salerno, J. B. Sauvan, Y. Sirois, A. Tarabini, A. Zabi, A. Zghiche, J.-L. Agram, J. Andrea, D. Apparu, D. Bloch, G. Bourgatte, J.-M. Brom, E. C. Chabert, C. Collard, D. Darej, J. -C. Fontaine, U. Goerlach, C. Grimault, A.-C. Le Bihan, E. Nibigira, P. Van Hove, E. Asilar, S. Beauceron, C. Bernet, G. Boudoul, C. Camen, A. Carle, N. Chanon, D. Contardo, P. Depasse, H. El Mamouni, J. Fay, S. Gascon, M. Gouzevitch, B. Ille, I. B. Laktineh, H. Lattaud, A. Lesauvage, M. Lethuillier, L. Mirabito, S. Perries, K. Shchablo, V. Sordini, L. Torterotot, G. Touquet, M. Vander Donckt, S. Viret, I. Lomidze, T. Toriashvili, Z. Tsamalaidze, V. Botta, L. Feld, K. Klein, M. Lipinski, D. Meuser, A. Pauls, M. P. Rauch, N. Röwert, J. Schulz, M. Teroerde, A. Dodonova, D. Eliseev, M. Erdmann, P. Fackeldey, B. Fischer, S. Ghosh, T. Hebbeker, K. Hoepfner, F. Ivone, H. Keller, L. Mastrolorenzo, M. Merschmeyer, A. Meyer, G. Mocellin, S. Mondal, S. Mukherjee, D. Noll, A. Novak, T. Pook, A. Pozdnyakov, Y. Rath, H. Reithler, J. Roemer, A. Schmidt, S. C. Schuler, A. Sharma, L. Vigilante, S. Wiedenbeck, S. Zaleski, C. Dziwok, G. Flügge, W. Haj Ahmad, O. Hlushchenko, T. Kress, A. Nowack, C. Pistone, O. Pooth, D. Roy, H. Sert, A. Stahl, T. Ziemons, H. Aarup Petersen, M. Aldaya Martin, P. Asmuss, I. Babounikau, S. Baxter, O. Behnke, A. Bermúdez Martínez, S. Bhattacharya, A. A. Bin Anuar, K. Borras, D. Brunner, A. Campbell, A. Cardini, C. Cheng, F. Colombina, S. Consuegra Rodríguez, G. Correia Silva, V. Danilov, M. De Silva, L. Didukh, G. Eckerlin, D. Eckstein, L. I. Estevez Banos, O. Filatov, E. Gallo, A. Geiser, A. Giraldi, A. Grohsjean, M. Guthoff, A. Jafari, N. Z. Jomhari, A. Kasem, M. Kasemann, H. Kaveh, C. Kleinwort, D. Krücker, W. Lange, J. Lidrych, K. Lipka, W. Lohmann, R. Mankel, I. -A. Melzer-Pellmann, M. Mendizabal Morentin, J. Metwally, A. B. Meyer, M. Meyer, J. Mnich, A. Mussgiller, Y. Otarid, D. Pérez Adán, D. Pitzl, A. Raspereza, B. Ribeiro Lopes, J. Rübenach, A. Saggio, A. Saibel, M. Savitskyi, M. Scham, V. Scheurer, P. Schütze, C. Schwanenberger, A. Singh, R. E. Sosa Ricardo, D. Stafford, N. Tonon, O. Turkot, M. Van De Klundert, R. Walsh, D. Walter, Y. Wen, K. Wichmann, L. Wiens, C. Wissing, S. Wuchterl, R. Aggleton, S. Albrecht, S. Bein, L. Benato, A. Benecke, P. Connor, K. De Leo, M. Eich, F. Feindt, A. Fröhlich, C. Garbers, E. Garutti, P. Gunnellini, J. Haller, A. Hinzmann, G. Kasieczka, R. Klanner, R. Kogler, T. Kramer, V. Kutzner, J. Lange, T. Lange, A. Lobanov, A. Malara, A. Nigamova, K. J. Pena Rodriguez, O. Rieger, P. Schleper, M. Schröder, J. Schwandt, D. Schwarz, J. Sonneveld, H. Stadie, G. Steinbrück, A. Tews, B. Vormwald, I. Zoi, J. Bechtel, T. Berger, E. Butz, R. Caspart, T. Chwalek, W. De Boer, A. Dierlamm, A. Droll, K. El Morabit, N. Faltermann, M. Giffels, J. o. Gosewisch, A. Gottmann, F. Hartmann, C. Heidecker, U. Husemann, P. Keicher, R. Koppenhöfer, S. Maier, M. Metzler, S. Mitra, Th. Müller, M. Neukum, A. Nürnberg, G. Quast, K. Rabbertz, J. Rauser, D. Savoiu, M. Schnepf, D. Seith, I. Shvetsov, H. J. Simonis, R. Ulrich, J. Van Der Linden, R. F. Von Cube, M. Wassmer, M. Weber, S. Wieland, R. Wolf, S. Wozniewski, S. Wunsch, G. Anagnostou, G. Daskalakis, T. Geralis, A. Kyriakis, A. Stakia, M. Diamantopoulou, D. Karasavvas, G. Karathanasis, P. Kontaxakis, C. K. Koraka, A. Manousakis-Katsikakis, A. Panagiotou, I. Papavergou, N. Saoulidou, K. Theofilatos, E. Tziaferi, K. Vellidis, E. Vourliotis, G. Bakas, K. Kousouris, I. Papakrivopoulos, G. Tsipolitis, A. Zacharopoulou, K. Adamidis, I. Bestintzanos, I. Evangelou, C. Foudas, P. Gianneios, P. Katsoulis, P. Kokkas, N. Manthos, I. Papadopoulos, J. Strologas, M. Csanád, K. Farkas, M. M. A. Gadallah, S. Lökös, P. Major, K. Mandal, A. Mehta, G. Pásztor, A. J. Rádl, O. Surányi, G. I. Veres, M. Bartók, G. Bencze, C. Hajdu, D. Horvath, F. Sikler, V. Veszpremi, G. Vesztergombi, S. Czellar, J. Karancsi, J. Molnar, Z. Szillasi, D. Teyssier, P. Raics, Z. L. Trocsanyi, B. Ujvari, T. Csorgo, F. Nemes, T. Novak, J. R. Komaragiri, D. Kumar, L. Panwar, P. C. Tiwari, S. Bansal, S. B. Beri, V. Bhatnagar, G. Chaudhary, S. Chauhan, N. Dhingra, R. Gupta, A. Kaur, M. Kaur, S. Kaur, P. Kumari, M. Meena, K. Sandeep, J. B. Singh, A. K. Virdi, A. Ahmed, A. Bhardwaj, B. C. Choudhary, M. Gola, S. Keshri, A. Kumar, M. Naimuddin, P. Priyanka, K. Ranjan, A. Shah, M. Bharti, R. Bhattacharya, S. Bhattacharya, D. Bhowmik, S. Dutta, S. Dutta, B. Gomber, M. Maity, P. Palit, P. K. Rout, G. Saha, B. Sahu, S. Sarkar, M. Sharan, B. Singh, S. Thakur, P. K. Behera, S. C. Behera, P. Kalbhor, A. Muhammad, R. Pradhan, P. R. Pujahari, A. Sharma, A. K. Sikdar, D. Dutta, V. Jha, V. Kumar, D. K. Mishra, K. Naskar, P. K. Netrakanti, L. M. Pant, P. Shukla, T. Aziz, S. Dugad, M. Kumar, G. B. Mohanty, U. Sarkar, S. Banerjee, R. Chudasama, M. Guchait, S. Karmakar, S. Kumar, G. Majumder, K. Mazumdar, S. Mukherjee, S. Bahinipati, C. Kar, P. Mal, T. Mishra, V. K. Muraleedharan Nair Bindhu, A. Nayak, P. Saha, N. Sur, S. K. Swain, D. Vats, K. Alpana, S. Dube, B. Kansal, A. Laha, S. Pandey, A. Rane, A. Rastogi, S. Sharma, H. Bakhshiansohi, E. Khazaie, M. Zeinali, S. Chenarani, S. M. Etesami, M. Khakzad, M. Mohammadi Najafabadi, M. Grunewald, M. Abbrescia, R. Aly, C. Aruta, A. Colaleo, D. Creanza, N. De Filippis, M. De Palma, A. Di Florio, A. Di Pilato, W. Elmetenawee, L. Fiore, A. Gelmi, M. Gul, G. Iaselli, M. Ince, S. Lezki, G. Maggi, M. Maggi, I. Margjeka, V. Mastrapasqua, J. A. Merlin, S. My, S. Nuzzo, A. Pellecchia, A. Pompili, G. Pugliese, A. Ranieri, G. Selvaggi, L. Silvestris, F. M. Simone, R. Venditti, P. Verwilligen, G. Abbiendi, C. Battilana, D. Bonacorsi, L. Borgonovi, L. Brigliadori, R. Campanini, P. Capiluppi, A. Castro, F. R. Cavallo, M. Cuffiani, G. M. Dallavalle, T. Diotalevi, F. Fabbri, A. Fanfani, P. Giacomelli, L. Giommi, C. Grandi, L. Guiducci, S. Lo Meo, L. Lunerti, S. Marcellini, G. Masetti, F. L. Navarria, A. Perrotta, F. Primavera, A. M. Rossi, T. Rovelli, G. P. Siroli, S. Albergo, S. Costa, A. Di Mattia, R. Potenza, A. Tricomi, C. Tuve, G. Barbagli, A. Cassese, R. Ceccarelli, V. Ciulli, C. Civinini, R. D’Alessandro, E. Focardi, G. Latino, P. Lenzi, M. Lizzo, M. Meschini, S. Paoletti, R. Seidita, G. Sguazzoni, L. Viliani, L. Benussi, S. Bianco, D. Piccolo, M. Bozzo, F. Ferro, R. Mulargia, E. Robutti, S. Tosi, A. Benaglia, G. Boldrini, F. Brivio, F. Cetorelli, V. Ciriolo, F. De Guio, M. E. Dinardo, P. Dini, S. Gennai, A. Ghezzi, P. Govoni, L. Guzzi, M. Malberti, S. Malvezzi, A. Massironi, D. Menasce, L. Moroni, M. Paganoni, D. Pedrini, B. S. Pinolini, S. Ragazzi, N. Redaelli, T. Tabarelli de Fatis, D. Valsecchi, D. Zuolo, S. Buontempo, F. Carnevali, N. Cavallo, A. De Iorio, F. Fabozzi, A. O. M. Iorio, L. Lista, S. Meola, P. Paolucci, B. Rossi, C. Sciacca, P. Azzi, N. Bacchetta, D. Bisello, P. Bortignon, A. Bragagnolo, R. Carlin, P. Checchia, T. Dorigo, U. Dosselli, F. Gasparini, U. Gasparini, G. Grosso, S. Y. Hoh, L. Layer, E. Lusiani, M. Margoni, A. T. Meneguzzo, J. Pazzini, M. Presilla, P. Ronchese, R. Rossin, F. Simonetto, G. Strong, M. Tosi, H. Yarar, M. Zanetti, P. Zotto, A. Zucchetta, G. Zumerle, C. Aime‘, A. Braghieri, S. Calzaferri, D. Fiorina, P. Montagna, S. P. Ratti, V. Re, C. Riccardi, P. Salvini, I. Vai, P. Vitulo, P. Asenov, G. M. Bilei, D. Ciangottini, L. Fanò, P. Lariccia, M. Magherini, G. Mantovani, V. Mariani, M. Menichelli, F. Moscatelli, A. Piccinelli, A. Rossi, A. Santocchia, D. Spiga, T. Tedeschi, P. Azzurri, G. Bagliesi, V. Bertacchi, L. Bianchini, T. Boccali, E. Bossini, R. Castaldi, M. A. Ciocci, V. D’Amante, R. Dell’Orso, M. R. Di Domenico, S. Donato, A. Giassi, F. Ligabue, E. Manca, G. Mandorli, A. Messineo, F. Palla, S. Parolia, G. Ramirez-Sanchez, A. Rizzi, G. Rolandi, S. Roy Chowdhury, A. Scribano, N. Shafiei, P. Spagnolo, R. Tenchini, G. Tonelli, N. Turini, A. Venturi, P. G. Verdini, M. Campana, F. Cavallari, D. Del Re, E. Di Marco, M. Diemoz, E. Longo, P. Meridiani, G. Organtini, F. Pandolfi, R. Paramatti, C. Quaranta, S. Rahatlou, C. Rovelli, F. Santanastasio, L. Soffi, R. Tramontano, N. Amapane, R. Arcidiacono, S. Argiro, M. Arneodo, N. Bartosik, R. Bellan, A. Bellora, J. Berenguer Antequera, C. Biino, N. Cartiglia, S. Cometti, M. Costa, R. Covarelli, N. Demaria, B. Kiani, F. Legger, C. Mariotti, S. Maselli, E. Migliore, E. Monteil, M. Monteno, M. M. Obertino, G. Ortona, L. Pacher, N. Pastrone, M. Pelliccioni, G. L. Pinna Angioni, M. Ruspa, K. Shchelina, F. Siviero, V. Sola, A. Solano, D. Soldi, A. Staiano, M. Tornago, D. Trocino, A. Vagnerini, S. Belforte, V. Candelise, M. Casarsa, F. Cossutti, A. Da Rold, G. Della Ricca, G. Sorrentino, F. Vazzoler, S. Dogra, C. Huh, B. Kim, D. H. Kim, G. N. Kim, J. Kim, J. Lee, S. W. Lee, C. S. Moon, Y. D. Oh, S. I. Pak, B. C. Radburn-Smith, S. Sekmen, Y. C. Yang, H. Kim, D. H. Moon, B. Francois, T. J. Kim, J. Park, S. Cho, S. Choi, Y. Go, B. Hong, K. Lee, K. S. Lee, J. Lim, J. Park, S. K. Park, J. Yoo, J. Goh, A. Gurtu, H. S. Kim, Y. Kim, J. Almond, J. H. Bhyun, J. Choi, S. Jeon, J. Kim, J. S. Kim, S. Ko, H. Kwon, H. Lee, S. Lee, B. H. Oh, M. Oh, S. B. Oh, H. Seo, U. K. Yang, I. Yoon, W. Jang, D. Y. Kang, Y. Kang, S. Kim, B. Ko, J. S. H. Lee, Y. Lee, I. C. Park, Y. Roh, M. S. Ryu, D. Song, Watson I.J., S. Yang, S. Ha, H. D. Yoo, M. Choi, Y. Jeong, H. Lee, Y. Lee, I. Yu, T. Beyrouthy, Y. Maghrbi, T. Torims, V. Veckalns, M. Ambrozas, A. Carvalho Antunes De Oliveira, A. Juodagalvis, A. Rinkevicius, G. Tamulaitis, N. Bin Norjoharuddeen, W. A. T. Wan Abdullah, M. N. Yusli, Z. Zolkapli, J. F. Benitez, A. Castaneda Hernandez, M. León Coello, J. A. Murillo Quijada, A. Sehrawat, L. Valencia Palomo, G. Ayala, H. Castilla-Valdez, E. De La Cruz-Burelo, I. Heredia-De La Cruz, R. Lopez-Fernandez, C. A. Mondragon Herrera, D. A. Perez Navarro, A. Sánchez Hernández, S. Carrillo Moreno, C. Oropeza Barrera, F. Vazquez Valencia, I. Pedraza, H. A. Salazar Ibarguen, C. Uribe Estrada, I. Bubanja, J. Mijuskovic, N. Raicevic, D. Krofcheck, S. Bheesette, P. H. Butler, A. Ahmad, M. I. Asghar, A. Awais, M. I. M. Awan, H. R. Hoorani, W. A. Khan, M. A. Shah, M. Shoaib, M. Waqas, V. Avati, L. Grzanka, M. Malawski, H. Bialkowska, M. Bluj, B. Boimska, M. Górski, M. Kazana, M. Szleper, P. Zalewski, K. Bunkowski, K. Doroba, A. Kalinowski, M. Konecki, J. Krolikowski, M. Walczak, M. Araujo, P. Bargassa, D. Bastos, A. Boletti, P. Faccioli, M. Gallinaro, J. Hollar, N. Leonardo, T. Niknejad, M. Pisano, J. Seixas, O. Toldaiev, J. Varela, P. Adzic, M. Dordevic, P. Milenovic, J. Milosevic, M. Aguilar-Benitez, J. Alcaraz Maestre, A. Álvarez Fernández, I. Bachiller, M. Barrio Luna, Cristina F. Bedoya, C. A. Carrillo Montoya, M. Cepeda, M. Cerrada, N. Colino, B. De La Cruz, A. Delgado Peris, J. P. Fernández Ramos, J. Flix, M. C. Fouz, O. Gonzalez Lopez, S. Goy Lopez, J. M. Hernandez, M. I. Josa, J. León Holgado, D. Moran, Á. Navarro Tobar, C. Perez Dengra, A. Pérez-Calero Yzquierdo, J. Puerta Pelayo, I. Redondo, L. Romero, S. Sánchez Navas, L. Urda Gómez, C. Willmott, J. F. de Trocóniz, R. Reyes-Almanza, B. Alvarez Gonzalez, J. Cuevas, C. Erice, J. Fernandez Menendez, S. Folgueras, I. Gonzalez Caballero, J. R. González Fernández, E. Palencia Cortezon, C. Ramón Álvarez, V. Rodríguez Bouza, A. Trapote, N. Trevisani, J. A. Brochero Cifuentes, I. J. Cabrillo, A. Calderon, J. Duarte Campderros, M. Fernandez, C. Fernandez Madrazo, P. J. Fernández Manteca, A. García Alonso, G. Gomez, C. Martinez Rivero, P. Martinez Ruiz del Arbol, F. Matorras, P. Matorras Cuevas, J. Piedra Gomez, C. Prieels, T. Rodrigo, A. Ruiz-Jimeno, L. Scodellaro, I. Vila, J. M. Vizan Garcia, M. K. Jayananda, B. Kailasapathy, D. U. J. Sonnadara, D. D. C. Wickramarathna, W. G. D. Dharmaratna, K. Liyanage, N. Perera, N. Wickramage, T. K. Aarrestad, D. Abbaneo, J. Alimena, E. Auffray, G. Auzinger, J. Baechler, P. Baillon, D. Barney, J. Bendavid, M. Bianco, A. Bocci, T. Camporesi, M. Capeans Garrido, G. Cerminara, S. S. Chhibra, M. Cipriani, L. Cristella, D. d’Enterria, A. Dabrowski, A. David, A. De Roeck, M. M. Defranchis, M. Deile, M. Dobson, M. Dünser, N. Dupont, A. Elliott-Peisert, N. Emriskova, F. Fallavollita, D. Fasanella, A. Florent, G. Franzoni, W. Funk, S. Giani, D. Gigi, K. Gill, F. Glege, L. Gouskos, M. Haranko, J. Hegeman, Y. Iiyama, V. Innocente, T. James, P. Janot, J. Kaspar, J. Kieseler, M. Komm, N. Kratochwil, C. Lange, S. Laurila, P. Lecoq, K. Long, C. Lourenço, L. Malgeri, S. Mallios, M. Mannelli, A. C. Marini, F. Meijers, S. Mersi, E. Meschi, F. Moortgat, M. Mulders, S. Orfanelli, L. Orsini, F. Pantaleo, L. Pape, E. Perez, M. Peruzzi, A. Petrilli, G. Petrucciani, A. Pfeiffer, M. Pierini, D. Piparo, M. Pitt, H. Qu, T. Quast, D. Rabady, A. Racz, G. Reales Gutiérrez, M. Rieger, M. Rovere, H. Sakulin, J. Salfeld-Nebgen, S. Scarfi, C. Schäfer, M. Selvaggi, A. Sharma, P. Silva, W. Snoeys, P. Sphicas, S. Summers, K. Tatar, V. R. Tavolaro, D. Treille, A. Tsirou, G. P. Van Onsem, J. Wanczyk, K. A. Wozniak, W. D. Zeuner, L. Caminada, A. Ebrahimi, W. Erdmann, R. Horisberger, Q. Ingram, H. C. Kaestli, D. Kotlinski, M. Missiroli, T. Rohe, K. Androsov, M. Backhaus, P. Berger, A. Calandri, N. Chernyavskaya, A. De Cosa, G. Dissertori, M. Dittmar, M. Donegà, C. Dorfer, F. Eble, K. Gedia, F. Glessgen, T. A. Gómez Espinosa, C. Grab, D. Hits, W. Lustermann, A.-M. Lyon, R. A. Manzoni, C. Martin Perez, M. T. Meinhard, F. Nessi-Tedaldi, J. Niedziela, F. Pauss, V. Perovic, S. Pigazzini, M. G. Ratti, M. Reichmann, C. Reissel, T. Reitenspiess, B. Ristic, D. Ruini, D. A. Sanz Becerra, M. Schönenberger, V. Stampf, J. Steggemann, R. Wallny, D. H. Zhu, C. Amsler, P. Bärtschi, C. Botta, D. Brzhechko, M. F. Canelli, K. Cormier, A. De Wit, R. Del Burgo, J. K. Heikkilä, M. Huwiler, W. Jin, A. Jofrehei, B. Kilminster, S. Leontsinis, S. P. Liechti, A. Macchiolo, P. Meiring, V. M. Mikuni, U. Molinatti, I. Neutelings, A. Reimers, P. Robmann, S. Sanchez Cruz, K. Schweiger, Y. Takahashi, C. Adloff, C. M. Kuo, W. Lin, A. Roy, T. Sarkar, S. S. Yu, L. Ceard, Y. Chao, K. F. Chen, P. H. Chen, W.-S. Hou, Y.y. Li, R.-S. Lu, E. Paganis, A. Psallidas, A. Steen, H. y. Wu, E. Yazgan, P. r. Yu, B. Asavapibhop, C. Asawatangtrakuldee, N. Srimanobhas, F. Boran, S. Damarseckin, Z. S. Demiroglu, F. Dolek, I. Dumanoglu, E. Eskut, Y. Guler, E. Gurpinar Guler, I. Hos, C. Isik, O. Kara, A. Kayis Topaksu, U. Kiminsu, G. Onengut, K. Ozdemir, A. Polatoz, A. E. Simsek, B. Tali, U. G. Tok, S. Turkcapar, I. S. Zorbakir, C. Zorbilmez, B. Isildak, G. Karapinar, K. Ocalan, M. Yalvac, B. Akgun, I. O. Atakisi, E. Gülmez, M. Kaya, O. Kaya, Ö. Özçelik, S. Tekten, E. A. Yetkin, A. Cakir, K. Cankocak, Y. Komurcu, S. Sen, S. Cerci, B. Kaynak, S. Ozkorucuklu, D. Sunar Cerci, B. Grynyov, L. Levchuk, D. Anthony, E. Bhal, S. Bologna, J. J. Brooke, A. Bundock, E. Clement, D. Cussans, H. Flacher, J. Goldstein, G. P. Heath, H. F. Heath, M.-L. Holmberg, L. Kreczko, B. Krikler, S. Paramesvaran, S. Seif El Nasr-Storey, V. J. Smith, N. Stylianou, K. Walkingshaw Pass, R. White, K. W. Bell, A. Belyaev, C. Brew, R. M. Brown, D. J. A. Cockerill, C. Cooke, K. V. Ellis, K. Harder, S. Harper, J. Linacre, K. Manolopoulos, D. M. Newbold, E. Olaiya, D. Petyt, T. Reis, T. Schuh, C. H. Shepherd-Themistocleous, I. R. Tomalin, T. Williams, R. Bainbridge, P. Bloch, S. Bonomally, J. Borg, S. Breeze, O. Buchmuller, V. Cepaitis, G. S. Chahal, D. Colling, P. Dauncey, G. Davies, M. Della Negra, S. Fayer, G. Fedi, G. Hall, M. H. Hassanshahi, G. Iles, J. Langford, L. Lyons, A.-M. Magnan, S. Malik, A. Martelli, D. G. Monk, J. Nash, M. Pesaresi, D. M. Raymond, A. Richards, A. Rose, E. Scott, C. Seez, A. Shtipliyski, A. Tapper, K. Uchida, T. Virdee, M. Vojinovic, N. Wardle, S. N. Webb, D. Winterbottom, K. Coldham, J. E. Cole, A. Khan, P. Kyberd, I. D. Reid, L. Teodorescu, S. Zahid, S. Abdullin, A. Brinkerhoff, B. Caraway, J. Dittmann, K. Hatakeyama, A. R. Kanuganti, B. McMaster, N. Pastika, M. Saunders, S. Sawant, C. Sutantawibul, J. Wilson, R. Bartek, A. Dominguez, R. Uniyal, A. M. Vargas Hernandez, A. Buccilli, S. I. Cooper, D. Di Croce, S. V. Gleyzer, C. Henderson, C. U. Perez, P. Rumerio, C. West, A. Akpinar, A. Albert, D. Arcaro, C. Cosby, Z. Demiragli, E. Fontanesi, D. Gastler, J. Rohlf, K. Salyer, D. Sperka, D. Spitzbart, I. Suarez, A. Tsatsos, S. Yuan, D. Zou, G. Benelli, B. Burkle, X. Coubez, D. Cutts, M. Hadley, U. Heintz, J. M. Hogan, G. Landsberg, K. T. Lau, M. Lukasik, J. Luo, M. Narain, S. Sagir, E. Usai, W. Y. Wong, X. Yan, D. Yu, W. Zhang, J. Bonilla, C. Brainerd, R. Breedon, M. Calderon De La Barca Sanchez, M. Chertok, J. Conway, P. T. Cox, R. Erbacher, G. Haza, F. Jensen, O. Kukral, R. Lander, M. Mulhearn, D. Pellett, B. Regnery, D. Taylor, Y. Yao, F. Zhang, M. Bachtis, R. Cousins, A. Datta, D. Hamilton, J. Hauser, M. Ignatenko, M. A. Iqbal, T. Lam, W. A. Nash, S. Regnard, D. Saltzberg, B. Stone, V. Valuev, K. Burt, Y. Chen, R. Clare, J. W. Gary, M. Gordon, G. Hanson, G. Karapostoli, O. R. Long, N. Manganelli, M. Olmedo Negrete, W. Si, S. Wimpenny, Y. Zhang, J. G. Branson, P. Chang, S. Cittolin, S. Cooperstein, N. Deelen, D. Diaz, J. Duarte, R. Gerosa, L. Giannini, D. Gilbert, J. Guiang, R. Kansal, V. Krutelyov, R. Lee, J. Letts, M. Masciovecchio, S. May, M. Pieri, B. V. Sathia Narayanan, V. Sharma, M. Tadel, A. Vartak, F. Würthwein, Y. Xiang, A. Yagil, N. Amin, C. Campagnari, M. Citron, A. Dorsett, V. Dutta, J. Incandela, M. Kilpatrick, J. Kim, B. Marsh, H. Mei, M. Oshiro, M. Quinnan, J. Richman, U. Sarica, F. Setti, J. Sheplock, D. Stuart, S. Wang, A. Bornheim, O. Cerri, I. Dutta, J. M. Lawhorn, N. Lu, J. Mao, H. B. Newman, T. Q. Nguyen, M. Spiropulu, J. R. Vlimant, C. Wang, S. Xie, Z. Zhang, R. Y. Zhu, J. Alison, S. An, M. B. Andrews, P. Bryant, T. Ferguson, A. Harilal, C. Liu, T. Mudholkar, M. Paulini, A. Sanchez, W. Terrill, J. P. Cumalat, W. T. Ford, A. Hassani, E. MacDonald, R. Patel, A. Perloff, C. Savard, K. Stenson, K. A. Ulmer, S. R. Wagner, J. Alexander, S. Bright-Thonney, Y. Cheng, D. J. Cranshaw, S. Hogan, J. Monroy, J. R. Patterson, D. Quach, J. Reichert, M. Reid, A. Ryd, W. Sun, J. Thom, P. Wittich, R. Zou, M. Albrow, M. Alyari, G. Apollinari, A. Apresyan, A. Apyan, S. Banerjee, L. A. T. Bauerdick, D. Berry, J. Berryhill, P. C. Bhat, K. Burkett, J. N. Butler, A. Canepa, G. B. Cerati, H. W. K. Cheung, F. Chlebana, M. Cremonesi, K. F. Di Petrillo, V. D. Elvira, Y. Feng, J. Freeman, Z. Gecse, L. Gray, D. Green, S. Grünendahl, O. Gutsche, R. M. Harris, R. Heller, T. C. Herwig, J. Hirschauer, B. Jayatilaka, S. Jindariani, M. Johnson, U. Joshi, T. Klijnsma, B. Klima, K. H. M. Kwok, S. Lammel, D. Lincoln, R. Lipton, T. Liu, C. Madrid, K. Maeshima, C. Mantilla, D. Mason, P. McBride, P. Merkel, S. Mrenna, S. Nahn, J. Ngadiuba, V. O’Dell, V. Papadimitriou, K. Pedro, C. Pena, O. Prokofyev, F. Ravera, A. Reinsvold Hall, L. Ristori, B. Schneider, E. Sexton-Kennedy, N. Smith, A. Soha, W. J. Spalding, L. Spiegel, J. Strait, L. Taylor, S. Tkaczyk, N. V. Tran, L. Uplegger, E. W. Vaandering, H. A. Weber, D. Acosta, P. Avery, D. Bourilkov, L. Cadamuro, V. Cherepanov, F. Errico, R. D. Field, D. Guerrero, B. M. Joshi, M. Kim, E. Koenig, J. Konigsberg, A. Korytov, K. H. Lo, K. Matchev, N. Menendez, G. Mitselmakher, A. Muthirakalayil Madhu, N. Rawal, D. Rosenzweig, S. Rosenzweig, K. Shi, J. Sturdy, J. Wang, E. Yigitbasi, X. Zuo, T. Adams, A. Askew, R. Habibullah, V. Hagopian, K. F. Johnson, R. Khurana, T. Kolberg, G. Martinez, H. Prosper, C. Schiber, O. Viazlo, R. Yohay, J. Zhang, M. M. Baarmand, S. Butalla, T. Elkafrawy, M. Hohlmann, R. Kumar Verma, D. Noonan, M. Rahmani, F. Yumiceva, M. R. Adams, H. Becerril Gonzalez, R. Cavanaugh, X. Chen, S. Dittmer, O. Evdokimov, C. E. Gerber, D. A. Hangal, D. J. Hofman, A. H. Merrit, C. Mills, G. Oh, T. Roy, S. Rudrabhatla, M. B. Tonjes, N. Varelas, J. Viinikainen, X. Wang, Z. Wu, Z. Ye, M. Alhusseini, K. Dilsiz, R. P. Gandrajula, O. K. Köseyan, J.-P. Merlo, A. Mestvirishvili, J. Nachtman, H. Ogul, Y. Onel, A. Penzo, C. Snyder, E. Tiras, O. Amram, B. Blumenfeld, L. Corcodilos, J. Davis, M. Eminizer, A. V. Gritsan, S. Kyriacou, P. Maksimovic, J. Roskes, M. Swartz, T. Á. Vámi, A. Abreu, J. Anguiano, C. Baldenegro Barrera, P. Baringer, A. Bean, A. Bylinkin, Z. Flowers, T. Isidori, S. Khalil, J. King, G. Krintiras, A. Kropivnitskaya, M. Lazarovits, C. Lindsey, J. Marquez, N. Minafra, M. Murray, M. Nickel, C. Rogan, C. Royon, R. Salvatico, S. Sanders, E. Schmitz, C. Smith, J. D. Tapia Takaki, Q. Wang, Z. Warner, J. Williams, G. Wilson, S. Duric, A. Ivanov, K. Kaadze, D. Kim, Y. Maravin, T. Mitchell, A. Modak, K. Nam, F. Rebassoo, D. Wright, E. Adams, A. Baden, O. Baron, A. Belloni, S. C. Eno, N. J. Hadley, S. Jabeen, R. G. Kellogg, T. Koeth, A. C. Mignerey, S. Nabili, C. Palmer, M. Seidel, A. Skuja, L. Wang, K. Wong, D. Abercrombie, G. Andreassi, R. Bi, S. Brandt, W. Busza, I. A. Cali, Y. Chen, M. D’Alfonso, J. Eysermans, C. Freer, G. Gomez-Ceballos, M. Goncharov, P. Harris, M. Hu, M. Klute, D. Kovalskyi, J. Krupa, Y.-J. Lee, B. Maier, C. Mironov, C. Paus, D. Rankin, C. Roland, G. Roland, Z. Shi, G. S. F. Stephans, J. Wang, Z. Wang, B. Wyslouch, R. M. Chatterjee, A. Evans, P. Hansen, J. Hiltbrand, Sh. Jain, M. Krohn, Y. Kubota, J. Mans, M. Revering, R. Rusack, R. Saradhy, N. Schroeder, N. Strobbe, M. A. Wadud, K. Bloom, M. Bryson, S. Chauhan, D. R. Claes, C. Fangmeier, L. Finco, F. Golf, C. Joo, I. Kravchenko, M. Musich, I. Reed, J. E. Siado, G. R. Snow, W. Tabb, F. Yan, A. G. Zecchinelli, G. Agarwal, H. Bandyopadhyay, L. Hay, I. Iashvili, A. Kharchilava, C. McLean, D. Nguyen, J. Pekkanen, S. Rappoccio, A. Williams, G. Alverson, E. Barberis, Y. Haddad, A. Hortiangtham, J. Li, G. Madigan, B. Marzocchi, D. M. Morse, V. Nguyen, T. Orimoto, A. Parker, L. Skinnari, A. Tishelman-Charny, T. Wamorkar, B. Wang, A. Wisecarver, D. Wood, S. Bhattacharya, J. Bueghly, Z. Chen, A. Gilbert, T. Gunter, K. A. Hahn, Y. Liu, N. Odell, M. H. Schmitt, M. Velasco, R. Band, R. Bucci, A. Das, N. Dev, R. Goldouzian, M. Hildreth, K. Hurtado Anampa, C. Jessop, K. Lannon, J. Lawrence, N. Loukas, L. Lutton, N. Marinelli, I. Mcalister, T. McCauley, C. Mcgrady, F. Meng, K. Mohrman, Y. Musienko, R. Ruchti, P. Siddireddy, A. Townsend, M. Wayne, A. Wightman, M. Wolf, M. Zarucki, L. Zygala, B. Bylsma, B. Cardwell, L. S. Durkin, B. Francis, C. Hill, M. Nunez Ornelas, K. Wei, B. L. Winer, B. R. Yates, F. M. Addesa, B. Bonham, P. Das, G. Dezoort, P. Elmer, A. Frankenthal, B. Greenberg, N. Haubrich, S. Higginbotham, A. Kalogeropoulos, G. Kopp, S. Kwan, D. Lange, M. T. Lucchini, D. Marlow, K. Mei, I. Ojalvo, J. Olsen, D. Stickland, C. Tully, S. Malik, S. Norberg, A. S. Bakshi, V. E. Barnes, R. Chawla, S. Das, L. Gutay, M. Jones, A. W. Jung, S. Karmarkar, M. Liu, G. Negro, N. Neumeister, G. Paspalaki, C. C. Peng, S. Piperov, A. Purohit, J. F. Schulte, M. Stojanovic, J. Thieman, F. Wang, R. Xiao, W. Xie, J. Dolen, N. Parashar, A. Baty, M. Decaro, S. Dildick, K. M. Ecklund, S. Freed, P. Gardner, F. J. M. Geurts, A. Kumar, W. Li, B. P. Padley, R. Redjimi, W. Shi, A. G. Stahl Leiton, S. Yang, L. Zhang, Y. Zhang, A. Bodek, P. de Barbaro, R. Demina, J. L. Dulemba, C. Fallon, T. Ferbel, M. Galanti, A. Garcia-Bellido, O. Hindrichs, A. Khukhunaishvili, E. Ranken, R. Taus, B. Chiarito, J. P. Chou, A. Gandrakota, Y. Gershtein, E. Halkiadakis, A. Hart, M. Heindl, O. Karacheban, I. Laflotte, A. Lath, R. Montalvo, K. Nash, M. Osherson, S. Salur, S. Schnetzer, S. Somalwar, R. Stone, S. A. Thayil, S. Thomas, H. Wang, H. Acharya, A. G. Delannoy, S. Fiorendi, S. Spanier, O. Bouhali, M. Dalchenko, A. Delgado, R. Eusebi, J. Gilmore, T. Huang, T. Kamon, H. Kim, S. Luo, S. Malhotra, R. Mueller, D. Overton, D. Rathjens, A. Safonov, N. Akchurin, J. Damgov, V. Hegde, S. Kunori, K. Lamichhane, S. W. Lee, T. Mengke, S. Muthumuni, T. Peltola, I. Volobouev, Z. Wang, A. Whitbeck, E. Appelt, S. Greene, A. Gurrola, W. Johns, A. Melo, H. Ni, K. Padeken, F. Romeo, P. Sheldon, S. Tuo, J. Velkovska, M. W. Arenton, B. Cox, G. Cummings, J. Hakala, R. Hirosky, M. Joyce, A. Ledovskoy, A. Li, C. Neu, B. Tannenwald, S. White, E. Wolfe, N. Poudyal, K. Black, T. Bose, C. Caillol, S. Dasu, I. De Bruyn, P. Everaerts, F. Fienga, C. Galloni, H. He, M. Herndon, A. Hervé, U. Hussain, A. Lanaro, A. Loeliger, R. Loveless, J. Madhusudanan Sreekala, A. Mallampalli, A. Mohammadi, D. Pinna, A. Savin, V. Shang, V. Sharma, W. H. Smith, D. Teague, S. Trembath-Reichert, W. Vetens, S. Afanasiev, V. Andreev, Yu. Andreev, T. Aushev, M. Azarkin, A. Babaev, A. Belyaev, V. Blinov, E. Boos, V. Borshch, D. Budkouski, V. Bunichev, M. Chadeeva, A. Dermenev, T. Dimova, I. Dremin, M. Dubinin, L. Dudko, Y. Dydyshka, V. Epshteyn, G. Gavrilov, V. Gavrilov, S. Gninenko, V. Golovtcov, N. Golubev, I. Golutvin, I. Gorbunov, V. Ivanchenko, Y. Ivanov, V. Kachanov, L. Kardapoltsev, V. Karjavine, A. Karneyeu, V. Kim, M. Kirakosyan, D. Kirpichnikov, M. Kirsanov, V. Klyukhin, O. Kodolova, D. Konstantinov, V. Korenkov, A. Kozyrev, N. Krasnikov, E. Kuznetsova, A. Lanev, O. Lukina, N. Lychkovskaya, V. Makarenko, A. Malakhov, V. Matveev, V. Mossolov, V. Murzin, A. Nikitenko, S. Obraztsov, V. Okhotnikov, V. Oreshkin, I. Ovtin, V. Palichik, P. Parygin, A. Pashenkov, V. Perelygin, M. Perfilov, G. Pivovarov, V. Popov, E. Popova, M. Savina, V. Savrin, D. Seitova, D. Selivanova, V. Shalaev, S. Shmatov, S. Shulha, Y. Skovpen, S. Slabospitskii, I. Smirnov, V. Smirnov, A. Snigirev, D. Sosnov, A. Spiridonov, A. Stepennov, V. Sulimov, E. Tcherniaev, A. Terkulov, O. Teryaev, D. Tlisov, M. Toms, A. Toropin, L. Uvarov, A. Uzunian, E. Vlasov, S. Volkov, A. Vorobyev, N. Voytishin, B. S. Yuldashev, A. Zarubin, E. Zhemchugov, I. Zhizhin, A. Zhokin, CMS Collaboration

**Affiliations:** 1 Yerevan Physics Institute, Yerevan, Armenia; 2 Institut für Hochenergiephysik, Vienna, Austria; 3 Universiteit Antwerpen, Antwerpen, Belgium; 4 Vrije Universiteit Brussel, Brussels, Belgium; 5 Université Libre de Bruxelles, Brussels, Belgium; 6 Ghent University, Ghent, Belgium; 7 Université Catholique de Louvain, Louvain-la-Neuve, Belgium; 8 Centro Brasileiro de Pesquisas Fisicas, Rio de Janeiro, Brazil; 9 Universidade do Estado do Rio de Janeiro, Rio de Janeiro, Brazil; 10 Universidade Estadual Paulista, Universidade Federal do ABC, São Paulo, Brazil; 11 Institute for Nuclear Research and Nuclear Energy, Bulgarian Academy of Sciences, Sofia, Bulgaria; 12 University of Sofia, Sofia, Bulgaria; 13 Beihang University, Beijing, China; 14 Department of Physics, Tsinghua University, Beijing, China; 15 Institute of High Energy Physics, Beijing, China; 16 State Key Laboratory of Nuclear Physics and Technology, Peking University, Beijing, China; 17 Sun Yat-Sen University, Guangzhou, China; 18 Institute of Modern Physics and Key Laboratory of Nuclear Physics and Ion-beam Application (MOE), Fudan University, Shanghai, China; 19 Zhejiang University, Hangzhou, Zhejiang China; 20 Universidad de Los Andes, Bogotá, Colombia; 21 Universidad de Antioquia, Medellín, Colombia; 22 Faculty of Electrical Engineering, Mechanical Engineering and Naval Architecture, University of Split, Split, Croatia; 23 Faculty of Science, University of Split, Split, Croatia; 24 Institute Rudjer Boskovic, Zagreb, Croatia; 25 University of Cyprus, Nicosia, Cyprus; 26 Charles University, Prague, Czech Republic; 27 Escuela Politecnica Nacional, Quito, Ecuador; 28 Universidad San Francisco de Quito, Quito, Ecuador; 29 Academy of Scientific Research and Technology of the Arab Republic of Egypt, Egyptian Network of High Energy Physics, Cairo, Egypt; 30 Center for High Energy Physics (CHEP-FU), Fayoum University, El-Fayoum, Egypt; 31 National Institute of Chemical Physics and Biophysics, Tallinn, Estonia; 32 Department of Physics, University of Helsinki, Helsinki, Finland; 33 Helsinki Institute of Physics, Helsinki, Finland; 34 Lappeenranta-Lahti University of Technology, Lappeenranta, Finland; 35 IRFU, CEA, Université Paris-Saclay, Gif-sur-Yvette, France; 36 Laboratoire Leprince-Ringuet, CNRS/IN2P3, Ecole Polytechnique, Institut Polytechnique de Paris, Palaiseau, France; 37 Université de Strasbourg, CNRS, IPHC UMR 7178, Strasbourg, France; 38 Institut de Physique des 2 Infinis de Lyon (IP2I ), Villeurbanne, France; 39 Georgian Technical University, Tbilisi, Georgia; 40 I. Physikalisches Institut, RWTH Aachen University, Aachen, Germany; 41 III. Physikalisches Institut A, RWTH Aachen University, Aachen, Germany; 42 III. Physikalisches Institut B, RWTH Aachen University, Aachen, Germany; 43 Deutsches Elektronen-Synchrotron, Hamburg, Germany; 44 University of Hamburg, Hamburg, Germany; 45 Karlsruher Institut fuer Technologie, Karlsruhe, Germany; 46 Institute of Nuclear and Particle Physics (INPP), NCSR Demokritos, Aghia Paraskevi, Greece; 47 National and Kapodistrian University of Athens, Athens, Greece; 48 National Technical University of Athens, Athens, Greece; 49 University of Ioánnina, Ioannina, Greece; 50 MTA-ELTE Lendület CMS Particle and Nuclear Physics Group, Eötvös Loránd University, Budapest, Hungary; 51 Wigner Research Centre for Physics, Budapest, Hungary; 52 Institute of Nuclear Research ATOMKI, Debrecen, Hungary; 53 Institute of Physics, University of Debrecen, Debrecen, Hungary; 54 Karoly Robert Campus, MATE Institute of Technology, Gyongyos, Hungary; 55 Indian Institute of Science (IISc), Bangalore, India; 56 Panjab University, Chandigarh, India; 57 University of Delhi, Delhi, India; 58 Saha Institute of Nuclear Physics, HBNI, Kolkata, India; 59 Indian Institute of Technology Madras, Madras, India; 60 Bhabha Atomic Research Centre, Mumbai, India; 61 Tata Institute of Fundamental Research-A, Mumbai, India; 62 Tata Institute of Fundamental Research-B, Mumbai, India; 63 National Institute of Science Education and Research, An OCC of Homi Bhabha National Institute, Bhubaneswar, Odisha India; 64 Indian Institute of Science Education and Research (IISER), Pune, India; 65 Isfahan University of Technology, Isfahan, Iran; 66 Institute for Research in Fundamental Sciences (IPM), Tehran, Iran; 67 University College Dublin, Dublin, Ireland; 68 INFN Sezione di Bari, Università di Bari, Politecnico di Bari, Bari, Italy; 69 INFN Sezione di Bologna, Università di Bologna, Bologna, Italy; 70 INFN Sezione di Catania, Università di Catania, Catania, Italy; 71 INFN Sezione di Firenze, Università di Firenze, Firenze, Italy; 72 INFN Laboratori Nazionali di Frascati, Frascati, Italy; 73 INFN Sezione di Genova, Università di Genova, Genoa, Italy; 74 INFN Sezione di Milano-Bicocca, Università di Milano-Bicocca, Milan, Italy; 75 INFN Sezione di Napoli, Università di Napoli ’Federico II’, Napoli, Italy; Università della Basilicata, Potenza, Italy; Università G. Marconi, Rome, Italy; 76 INFN Sezione di Padova, Università di Padova, Padova, Italy; Università di Trento, Trento, Italy; 77 INFN Sezione di Pavia, Università di Pavia, Pavia, Italy; 78 INFN Sezione di Perugia, Università di Perugia, Perugia, Italy; 79 INFN Sezione di Pisa, Università di Pisa, Scuola Normale Superiore di Pisa, Pisa, Italy; Università di Siena, Siena, Italy; 80 INFN Sezione di Roma, Sapienza Università di Roma, Rome, Italy; 81 INFN Sezione di Torino, Università di Torino, Torino, Italy; Università del Piemonte Orientale, Novara, Italy; 82 INFN Sezione di Trieste, Università di Trieste, Trieste, Italy; 83 Kyungpook National University, Daegu, Korea; 84 Chonnam National University, Institute for Universe and Elementary Particles, Kwangju, Korea; 85 Hanyang University, Seoul, Korea; 86 Korea University, Seoul, Korea; 87 Department of Physics, Kyung Hee University, Seoul, Korea; 88 Sejong University, Seoul, Korea; 89 Seoul National University, Seoul, Korea; 90 University of Seoul, Seoul, Korea; 91 Department of Physics, Yonsei University, Seoul, Korea; 92 Sungkyunkwan University, Suwon, Korea; 93 College of Engineering and Technology, American University of the Middle East (AUM), Dasman, Kuwait; 94 Riga Technical University, Riga, Latvia; 95 Vilnius University, Vilnius, Lithuania; 96 National Centre for Particle Physics, Universiti Malaya, Kuala Lumpur, Malaysia; 97 Universidad de Sonora (UNISON), Hermosillo, Mexico; 98 Centro de Investigacion y de Estudios Avanzados del IPN, Mexico City, Mexico; 99 Universidad Iberoamericana, Mexico City, Mexico; 100 Benemerita Universidad Autonoma de Puebla, Puebla, Mexico; 101 University of Montenegro, Podgorica, Montenegro; 102 University of Auckland, Auckland, New Zealand; 103 University of Canterbury, Christchurch, New Zealand; 104 National Centre for Physics, Quaid-I-Azam University, Islamabad, Pakistan; 105 AGH University of Science and Technology Faculty of Computer Science, Electronics and Telecommunications, Kraków, Poland; 106 National Centre for Nuclear Research, Swierk, Poland; 107 Institute of Experimental Physics, Faculty of Physics, University of Warsaw, Warsaw, Poland; 108 Laboratório de Instrumentação e Física Experimental de Partículas, Lisbon, Portugal; 109 VINCA Institute of Nuclear Sciences, University of Belgrade, Belgrade, Serbia; 110 Centro de Investigaciones Energéticas Medioambientales y Tecnológicas (CIEMAT), Madrid, Spain; 111 Universidad Autónoma de Madrid, Madrid, Spain; 112 Universidad de Oviedo, Instituto Universitario de Ciencias y Tecnologías Espaciales de Asturias (ICTEA), Oviedo, Spain; 113 Instituto de Física de Cantabria (IFCA), CSIC-Universidad de Cantabria, Santander, Spain; 114 University of Colombo, Colombo, Sri Lanka; 115 Department of Physics, University of Ruhuna, Matara, Sri Lanka; 116 CERN, European Organization for Nuclear Research, Geneva, Switzerland; 117 Paul Scherrer Institut, Villigen, Switzerland; 118 ETH Zurich-Institute for Particle Physics and Astrophysics (IPA), Zurich, Switzerland; 119 Universität Zürich, Zurich, Switzerland; 120 National Central University, Chung-Li, Taiwan; 121 National Taiwan University (NTU), Taipei, Taiwan; 122 Department of Physics, Faculty of Science, Chulalongkorn University, Bangkok, Thailand; 123 Physics Department, Science and Art Faculty, Çukurova University, Adana, Turkey; 124 Physics Department, Middle East Technical University, Ankara, Turkey; 125 Bogazici University, Istanbul, Turkey; 126 Istanbul Technical University, Istanbul, Turkey; 127 Istanbul University, Istanbul, Turkey; 128 Institute for Scintillation Materials of National Academy of Science of Ukraine, Kharkiv, Ukraine; 129 National Science Centre, Kharkiv Institute of Physics and Technology, Kharkiv, Ukraine; 130 University of Bristol, Bristol, UK; 131 Rutherford Appleton Laboratory, Didcot, UK; 132 Imperial College, London, UK; 133 Brunel University, Uxbridge, UK; 134 Baylor University, Waco, TX USA; 135 Catholic University of America, Washington, DC USA; 136 The University of Alabama, Tuscaloosa, AL USA; 137 Boston University, Boston, MA USA; 138 Brown University, Providence, RI USA; 139 University of California, Davis, Davis, CA USA; 140 University of California, Los Angeles, CA USA; 141 University of California, Riverside, Riverside, CA USA; 142 University of California, San Diego, La Jolla, CA USA; 143 Department of Physics, University of California, Santa Barbara, Santa Barbara, CA USA; 144 California Institute of Technology, Pasadena, CA USA; 145 Carnegie Mellon University, Pittsburgh, PA USA; 146 University of Colorado Boulder, Boulder, CO USA; 147 Cornell University, Ithaca, NY USA; 148 Fermi National Accelerator Laboratory, Batavia, IL USA; 149 University of Florida, Gainesville, FL USA; 150 Florida State University, Tallahassee, FL USA; 151 Florida Institute of Technology, Melbourne, FL USA; 152 University of Illinois at Chicago (UIC), Chicago, IL USA; 153 The University of Iowa, Iowa City, IA USA; 154 Johns Hopkins University, Baltimore, MD USA; 155 The University of Kansas, Lawrence, KS USA; 156 Kansas State University, Manhattan, KS USA; 157 Lawrence Livermore National Laboratory, Livermore, CA USA; 158 University of Maryland, College Park, MD USA; 159 Massachusetts Institute of Technology, Cambridge, MA USA; 160 University of Minnesota, Minneapolis, MN USA; 161 University of Nebraska-Lincoln, Lincoln, NE USA; 162 State University of New York at Buffalo, Buffalo, NY USA; 163 Northeastern University, Boston, MA USA; 164 Northwestern University, Evanston, IL USA; 165 University of Notre Dame, Notre Dame, IN USA; 166 The Ohio State University, Columbus, OH USA; 167 Princeton University, Princeton, NJ USA; 168 University of Puerto Rico, Mayaguez, PR USA; 169 Purdue University, West Lafayette, IN USA; 170 Purdue University Northwest, Hammond, IN USA; 171 Rice University, Houston, TX USA; 172 University of Rochester, Rochester, NY USA; 173 Rutgers, The State University of New Jersey, Piscataway, NJ USA; 174 University of Tennessee, Knoxville, TN USA; 175 Texas A &M University, College Station, TX USA; 176 Texas Tech University, Lubbock, TX USA; 177 Vanderbilt University, Nashville, TN USA; 178 University of Virginia, Charlottesville, VA USA; 179 Wayne State University, Detroit, MI USA; 180 University of Wisconsin-Madison, Madison, WI USA; 181 Authors affiliated with an institute or an international laboratory covered by a cooperation agreement with CERN, Geneva, Switzerland; 182 TU Wien, Vienna, Austria; 183 Institute of Basic and Applied Sciences, Faculty of Engineering, Arab Academy for Science, Technology and Maritime Transport, Alexandria, Egypt; 184 Université Libre de Bruxelles, Brussels, Belgium; 185 Universidade Estadual de Campinas, Campinas, Brazil; 186 Federal University of Rio Grande do Sul, Porto Alegre, Brazil; 187 University of Chinese Academy of Sciences, Beijing, China; 188 UFMS, Nova Andradina, Brazil; 189 Nanjing Normal University Department of Physics, Nanjing, China; 190 The University of Iowa, Iowa City, IA USA; 191 University of Chinese Academy of Sciences, Beijing, China; 192 an institute or an international laboratory covered by a cooperation agreement with CERN, Geneva, Switzerland; 193 Helwan University, Cairo, Egypt; 194 Zewail City of Science and Technology, Zewail, Egypt; 195 British University in Egypt, Cairo, Egypt; 196 Purdue University, West Lafayette, IN USA; 197 Université de Haute Alsace, Mulhouse, France; 198 Tbilisi State University, Tbilisi, Georgia; 199 Erzincan Binali Yildirim University, Erzincan, Turkey; 200 CERN, European Organization for Nuclear Research, Geneva, Switzerland; 201 III. Physikalisches Institut A, RWTH Aachen University, Aachen, Germany; 202 University of Hamburg, Hamburg, Germany; 203 Isfahan University of Technology, Isfahan, Iran; 204 Brandenburg University of Technology, Cottbus, Germany; 205 Physics Department, Faculty of Science, Assiut University, Assiut, Egypt; 206 Karoly Robert Campus, MATE Institute of Technology, Gyongyos, Hungary; 207 Institute of Physics, University of Debrecen, Debrecen, Hungary; 208 Institute of Nuclear Research ATOMKI, Debrecen, Hungary; 209 MTA-ELTE Lendület CMS Particle and Nuclear Physics Group, Eötvös Loránd University, Budapest, Hungary; 210 Wigner Research Centre for Physics, Budapest, Hungary; 211 G.H.G. Khalsa College, Ludhiana, Punjab India; 212 Shoolini University, Solan, India; 213 University of Hyderabad, Hyderabad, India; 214 University of Visva-Bharati, Santiniketan, India; 215 Indian Institute of Technology (IIT), Mumbai, India; 216 IIT Bhubaneswar, Bhubaneswar, India; 217 Institute of Physics, Bhubaneswar, India; 218 Deutsches Elektronen-Synchrotron, Hamburg, Germany; 219 Sharif University of Technology, Tehran, Iran; 220 Department of Physics, University of Science and Technology of Mazandaran, Behshahr, Iran; 221 Italian National Agency for New Technologies, Energy and Sustainable Economic Development, Bologna, Italy; 222 Centro Siciliano di Fisica Nucleare e di Struttura Della Materia, Catania, Italy; 223 Università di Napoli ’Federico II’, Naples, Italy; 224 Consiglio Nazionale delle Ricerche-Istituto Officina dei Materiali, Perugia, Italy; 225 Consejo Nacional de Ciencia y Tecnología, Mexico City, Mexico; 226 IRFU, CEA, Université Paris-Saclay, Gif-sur-Yvette, France; 227 Faculty of Physics, University of Belgrade, Belgrade, Serbia; 228 Trincomalee Campus, Eastern University, Sri Lanka, Nilaveli, Sri Lanka; 229 INFN Sezione di Pavia, Università di Pavia, Pavia, Italy; 230 National and Kapodistrian University of Athens, Athens, Greece; 231 Ecole Polytechnique Fédérale Lausanne, Lausanne, Switzerland; 232 Universität Zürich, Zurich, Switzerland; 233 Stefan Meyer Institute for Subatomic Physics, Vienna, Austria; 234 Laboratoire d’Annecy-le-Vieux de Physique des Particules, IN2P3-CNRS, Annecy-le-Vieux, France; 235 Şırnak University, Sirnak, Turkey; 236 Near East University, Research Center of Experimental Health Science, Mersin, Turkey; 237 Konya Technical University, Konya, Turkey; 238 Faculty of Engineering, Istanbul University-Cerrahpasa, Istanbul, Turkey; 239 Izmir Bakircay University, Izmir, Turkey; 240 Adiyaman University, Adiyaman, Turkey; 241 Yildiz Technical University, Istanbul, Turkey; 242 Izmir Institute of Technology, Izmir, Turkey; 243 Necmettin Erbakan University, Konya, Turkey; 244 Bozok Universitetesi Rektörlügü, Yozgat, Turkey; 245 Marmara University, Istanbul, Turkey; 246 Milli Savunma University, Istanbul, Turkey; 247 Kafkas University, Kars, Turkey; 248 Istanbul Bilgi University, Istanbul, Turkey; 249 Hacettepe University, Ankara, Turkey; 250 Rutherford Appleton Laboratory, Didcot, United Kingdom; 251 Vrije Universiteit Brussel, Brussels, Belgium; 252 School of Physics and Astronomy, University of Southampton, Southampton, UK; 253 IPPP Durham University, Durham, UK; 254 Monash University, Faculty of Science, Clayton, Australia; 255 Università di Torino, Turin, Italy; 256 Bethel University, St. Paul, MN USA; 257 Karamanoğlu Mehmetbey University, Karaman, Turkey; 258 California Institute of Technology, Pasadena, CA USA; 259 Ain Shams University, Cairo, Egypt; 260 Bingol University, Bingol, Turkey; 261 Georgian Technical University, Tbilisi, Georgia; 262 Sinop University, Sinop, Turkey; 263 Erciyes University, Kayseri, Turkey; 264 Texas A &M University at Qatar, Doha, Qatar; 265 Kyungpook National University, Daegu, Korea; 266 another institute or international laboratory covered by a cooperation agreement with CERN, Geneva, Switzerland; 267 Yerevan Physics Institute, Yerevan, Armenia; 268 University of Florida, Gainesville, FL USA; 269 Imperial College, London, UK; 270 Institute of Nuclear Physics of the Uzbekistan Academy of Sciences, Tashkent, Uzbekistan; 271 CERN, 1211 Geneva 23, Switzerland

## Abstract

The double differential cross sections of the Drell–Yan lepton pair ($\ell ^+\ell ^-$, dielectron or dimuon) production are measured as functions of the invariant mass $m_{\ell \ell }$, transverse momentum $p_{\textrm{T}} (\ell \ell )$, and $\varphi ^{*}_{\eta }$. The $\varphi ^{*}_{\eta }$ observable, derived from angular measurements of the leptons and highly correlated with $p_{\textrm{T}} (\ell \ell )$, is used to probe the low-$p_{\textrm{T}} (\ell \ell )$ region in a complementary way. Dilepton masses up to 1$\,\text {Te\hspace{-.08em}V}$ are investigated. Additionally, a measurement is performed requiring at least one jet in the final state. To benefit from partial cancellation of the systematic uncertainty, the ratios of the differential cross sections for various $m_{\ell \ell }$ ranges to those in the Z mass peak interval are presented. The collected data correspond to an integrated luminosity of 36.3$\,\text {fb}^{-1}$ of proton–proton collisions recorded with the CMS detector at the LHC at a centre-of-mass energy of 13$\,\text {Te\hspace{-.08em}V}$. Measurements are compared with predictions based on perturbative quantum chromodynamics, including soft-gluon resummation.

## Introduction

The Drell–Yan (DY) production of charged-lepton pairs in hadronic collisions [1] provides important insights into the partonic structure of hadrons and the evolution of the parton distribution functions (PDFs). At leading order (LO) in perturbative quantum chromodynamics (pQCD), the DY process is described in terms of an *s*-channel $Z/\gamma ^*$ exchange process convolved with collinear quark and antiquark parton distribution functions of the proton. At LO, the lepton pair transverse momentum $p_{\textrm{T}} (\ell \ell )$, corresponding to the exchanged boson transverse momentum, is equal to zero. At higher orders, initial-state QCD radiation gives rise to a sizable $p_{\textrm{T}} (\ell \ell )$. Whereas the spectrum for large $p_{\textrm{T}} (\ell \ell )$ values is expected to be described through fixed-order calculations in pQCD, at small values ($p_{\textrm{T}} < {\mathcal {O}}(m_{\ell \ell })$), where $m_{\ell \ell }$ is the invariant mass of the lepton pair, soft-gluon resummation to all orders is required [2, 3]. In addition, the low-$p_{\textrm{T}} (\ell \ell )$ region also includes the effects of the intrinsic transverse motion of the partons in the colliding hadrons that has to be extracted from data and parameterized. The resummation functions are universal and obey renormalisation group equations, predicting a simple scale dependence in the leading logarithmic approximation, where the scale is given by $m_{\ell \ell }$. Therefore, measuring the $p_{\textrm{T}} (\ell \ell )$ spectrum in a wide $m_{\ell \ell }$ range tests the validity of the resummation approach and the precision of different predictions. Calculations for inclusive DY production as a function of $m_{\ell \ell }$ and $p_{\textrm{T}} (\ell \ell )$ are available up to next-to-next-to-leading order (NNLO) in pQCD [4–7]. Soft-gluon resummation can be computed analytically, either in transverse momentum dependent parton distributions (TMD) or in parton showers of Monte Carlo (MC) event generators matched with matrix element calculations [8–15].

The $p_{\textrm{T}} (\ell \ell )$ resolution is dominated by the uncertainties in the magnitude of the transverse momenta of the leptons, whereas the measurement precision of the lepton angle does not contribute significantly. The kinematic quantity $\varphi ^{*}_{\eta }$ [16–18], derived from these lepton angles, is defined by the equation:

1 $$\begin{aligned} \varphi ^{*}_{\eta } \equiv \tan \left( \frac{\pi - \varDelta \varphi }{2}\right) \, \sin (\theta ^*_{\eta }). \end{aligned}$$

The variable $\varDelta \varphi$ is the opening angle between the leptons in the plane transverse to the beam axis. The variable $\theta ^*_{\eta }$ is the scattering angle of the dileptons with respect to the beam in the longitudinally boosted frame where the leptons are back to back. It is related to the pseudorapidities of the oppositely charged leptons by the relation $\cos (\theta ^*_{\eta }) = \tanh \left[ (\eta ^{-} - \eta ^{+})/2\right]$. The variable $\varphi ^{*}_{\eta }$, by construction greater than zero, is closely related to the normalized transverse momentum $p_{\textrm{T}} (\ell \ell )/m_{\ell \ell }$ [16]. Since $\varphi ^{*}_{\eta }$ depends only on angular variables, its resolution is significantly better than that of the transverse momentum, especially at low-$p_{\textrm{T}} (\ell \ell )$ values, but its interpretation in terms of initial-state radiation (ISR) is not as direct as that of $p_{\textrm{T}} (\ell \ell )$.

The DY process in the presence of one jet is a complementary way to investigate the initial-state QCD radiations. The requirement of a minimal transverse momentum associated with this jet is reflected in the $p_{\textrm{T}} (\ell \ell )$ distribution by momentum conservation. When more hadronic activity than a single jet is present in the events, the transverse momentum balance between the leading jet and the lepton pair has a broad distribution. As a consequence, the full $p_{\textrm{T}} (\ell \ell )$ spectrum in the presence of jets brings additional information, since at small values it is sensitive to numerous hard QCD radiations. Furthermore, DY production in association with at least one jet also brings up contributions where virtual partons acquire transverse momentum, whose collinear radiations will have a significant angle with respect to the beam, which contributes as a component of the final $p_{\textrm{T}} (\ell \ell )$.

This paper presents a DY differential cross section measurement in bins of $m_{\ell \ell }$, over the range of 50$\,\text {Ge\hspace{-.08em}V}$ to 1$\,\text {Te\hspace{-.08em}V}$, as functions of $p_{\textrm{T}} (\ell \ell )$ and $\varphi ^{*}_{\eta }$ for inclusive DY production, and in events with at least one jet as a function of $p_{\textrm{T}} (\ell \ell )$. The data were collected in 2016 with the CMS detector at the CERN LHC, corresponding to an integrated luminosity of 36.3$\,\text {fb}^{-1}$ of proton–proton (pp) collisions at a centre-of-mass energy of $\sqrt{s} = 13\,\text {Te\hspace{-.08em}V}$. To reduce the uncertainties, the measured cross sections combine measurements of separately extracted cross sections for the electron and the muon channels. The measurements presented in this paper are extensively discussed in Ref. [19].

Complementary measurements of the DY process have been performed recently by the CMS, ATLAS, and LHCb Collaborations at the CERN LHC [20–38] and by the CDF and D0 Collaborations at the Fermilab Tevatron [39–45]. The cross section measurements presented in this paper extend the mass range below and above the Z boson resonance with respect to the previous CMS measurements of $p_{\textrm{T}} (\ell \ell )$ dependence.

The outline of this paper is the following: in Sect. 2 a brief description of the CMS detector is given. In Sect. 3 the selection criteria of the measurement are described. The simulation samples used in the measurement are described in Sect. 4. Section 5 explains the details of the unfolding procedure and the systematic uncertainties are given in Sect. 6. Theory predictions used for comparison with the measurements are described in Sect. 7. The results are presented in Sect. 8 and a summary of the paper is given in Sect. 9.

## The CMS detector

The central feature of the CMS apparatus is a superconducting solenoid of $6\,{\textrm{m}}$ internal diameter, providing a magnetic field of $3.8\,{\textrm{T}}$. Within the solenoid volume are a silicon pixel and strip tracker, a lead tungstate crystal electromagnetic calorimeter (ECAL), and a brass and scintillator hadron calorimeter (HCAL), each composed of a barrel and two endcap sections. Forward calorimeters extend the $\eta$ coverage provided by the barrel and endcap detectors. Muons are detected in gas-ionization chambers made of detection planes using three technologies: drift tubes, cathode strip chambers, and resistive plate chambers, embedded in the steel flux-return yoke outside the solenoid.

The global event reconstruction (also called particle-flow event reconstruction [46]) reconstructs and identifies each individual particle in an event, with an optimized combination of all subdetector information. In this process, the identification of the particle type (photon, electron, muon, charged or neutral hadron) plays an important role in the determination of the particle direction and energy.

Electrons are identified as a primary charged particle track and potentially many ECAL energy clusters corresponding to this track extrapolation to the ECAL and to possible bremsstrahlung photons emitted along the way. The electron momenta are estimated by combining energy measurements in the ECAL with momentum measurements in the tracker [47]. The momentum resolution for electrons with $p_{\textrm{T}} \approx 45\,\text {Ge\hspace{-.08em}V}$ from $\text {Z} \rightarrow \text {e} \text {e}$ decays ranges from 1.7 to 4.5%. It is better in the barrel region than in the endcaps, and also depends on the bremsstrahlung energy emitted by the electron as it traverses the material in front of the ECAL.

Muons are identified as tracks in the central tracker consistent with either a track or several hits in the muon system, and associated with calorimeter deposits compatible with the muon hypothesis. The reconstructed muon global track, for muons with $20<p_{\textrm{T}} < 100\,\text {Ge\hspace{-.08em}V}$, has a relative transverse momentum resolution of 1.3–2.0% in the barrel and better than 6% in the endcaps. The $p_{\textrm{T}}$ resolution in the barrel is better than 10% for muons with $p_{\textrm{T}}$ up to 1$\,\text {Te\hspace{-.08em}V}$ [48]. The resolution is further improved with corrections derived from the Z mass distribution [49].

Charged hadrons are identified as charged particle tracks not identified as electrons or as muons. Finally, neutral hadrons are identified as HCAL energy clusters not linked to any charged-hadron trajectory, or as a combined ECAL and HCAL energy excess with respect to the expected charged-hadron energy deposit. For each event, hadronic jets are clustered from these reconstructed particles using the infrared- and collinear-safe anti-$k_{\textrm{T}}$ algorithm [50, 51] with a distance parameter of 0.4. Jet momentum is determined as the vectorial sum of all particle momenta in the jet, typically within 5–10% of the true momentum over the entire $p_{\textrm{T}}$ spectrum and detector acceptance.

The primary vertex (PV) is taken to be the vertex corresponding to the hardest scattering in the event, evaluated using tracking information alone, as described in Section 9.4.1 of Ref. [52].

Events of interest are selected using a two-tiered trigger system. The first level (L1), composed of custom hardware processors, uses information from the calorimeters and muon detectors to select events at a rate of around $100\,{\textrm{kHz}}$ within a fixed latency of about 4 $\,\upmu \text {s}$ [53]. The second level, known as the high-level trigger, consists of a farm of processors running a version of the full event reconstruction software optimized for fast processing, and reduces the event rate to around $1\,{\textrm{kHz}}$ before data storage [54].

A more detailed description of the CMS detector is reported in Ref. [55], together with a definition of the coordinate system used and the relevant kinematic variables.

## Event selection

The initial event selection requires a dielectron trigger with a $p_{\textrm{T}}$ threshold of 23 and 12$\,\text {Ge\hspace{-.08em}V}$ on the two leading electrons in the electron channel. In the muon channel we require a dimuon trigger with $p_{\textrm{T}}$ thresholds of 18 and 7$\,\text {Ge\hspace{-.08em}V}$ or a single-muon trigger with a $p_{\textrm{T}}$ threshold of 24$\,\text {Ge\hspace{-.08em}V}$. The final selection is restricted to the region where the triggers are fully efficient: $p_{\textrm{T}} > 25\,\text {Ge\hspace{-.08em}V}$ for the leading lepton, $p_{\textrm{T}} > 20\,\text {Ge\hspace{-.08em}V}$ for the subleading lepton and $|\eta | < 2.4$ for both channels.

An event must contain exactly two isolated leptons of the same flavour (with the isolation criteria as detailed in Ref. [26]). In addition the two leptons must have opposite charges. Events with a third lepton with $p_{\textrm{T}}$ greater than 10$\,\text {Ge\hspace{-.08em}V}$ and $|\eta |<2.4$ are vetoed.

Due to the high instantaneous luminosity of the LHC, additional proton–proton interactions occur during the same bunch crossing (pileup) that contribute additional overlapping tracks and energy deposits in the event, and result in an apparent increase of jet momenta. To mitigate this effect, tracks identified as originating from pileup vertices are discarded and an offset correction is applied to correct for the remaining neutral pileup contributions [56]. The two identified leptons can be reconstructed as jets. Those jets are disregarded by requiring a separation, $\varDelta R = \sqrt{\smash [b]{(\varDelta \eta )^2 + (\varDelta \phi )^2}}$, between the reconstructed jets and these lepton candidates to be larger than 0.4.

To suppress the contamination of jets coming from pileup, a multivariate discriminant is used. The pileup contamination is also reduced by the choice of the final selection: jets are required to have a minimum transverse momentum of 30$\,\text {Ge\hspace{-.08em}V}$ and, to ensure high-quality track information, they are limited to a rapidity range of $|y |< 2.4$.

To reduce the ${\text {t}} {\bar{\text {t}}}$ background, events containing one or more b tagged jets are vetoed. The medium discrimination working point of the combined secondary vertex b tagging algorithm [57] is used. The effect on the signal is small and is corrected for in the unfolding procedure.

The effects of finite detector resolution and selection efficiency are corrected by using the unfolding procedure described in Sect. 5. Scale factors are applied to the simulation used for the unfolding, to correct for differences with respect to the data in the efficiencies of the different selections: trigger, lepton identification, lepton isolation, and b-tagged jet veto. For the trigger, the factor is given as a function of $|\eta |$ of the two leptons and is applied once per lepton pair. The value of the scale factor is close to one. When dealing with the identification and isolation efficiencies, the scale factor is given per lepton as a function of its $p_{\textrm{T}}$ and $|\eta |$, and applied to each of the two selected leptons [26].

## Simulated samples and backgrounds

For the simulation of the $\text {Z}/\gamma ^*$ process (including the ${{\uptau }} ^+{{\uptau }} ^-$ background), a sample is generated with $\textsc {MadGraph} {}5\_a\textsc {mc@nlo}$[58] version 5.2.2.2 (shortened to mg5_amc) using the FxFx jet merging scheme [59]. The parton shower, hadronization, and QED final-state radiation (FSR) are calculated with pythia 8.212 [60] using the CUETP8M1 tune [61]. The matrix element calculations include $\text {Z}/\gamma ^* + 0,1,2$ jets at next-to-leading order (NLO), giving an LO accuracy for $\text {Z}/\gamma ^* + 3$ jets. The NLO NNPDF 3.0 [62] is used for the matrix element calculation. In control plots and when comparing to the measurement, this prediction is normalized to the cross section obtained directly from the generator, 1977 pb per lepton channel (for $m_{\ell \ell } >50\,\text {Ge\hspace{-.08em}V}$).

Other processes that can give a final state with two oppositely charged same-flavour leptons are $\text {W} \text {W}$, $\text {W} \text {Z}$, $\text {Z} \text {Z}$, $\gamma \gamma$, ${{\text {t}} {}{\bar{\text {t}}}}$ pairs, and single top quark production. The ${{\text {t}} {}{\bar{\text {t}}}}$ and single top backgrounds are generated at NLO using the powheg version 2 [63–66] interfaced to pythia 8. Background samples corresponding to diboson electroweak production (denoted VV in the figure legends) [67] are generated at NLO with powheg interfaced to pythia 8 ($\text {W} \text {W}$) or at LO with pythia 8 alone ($\text {W} \text {Z}$ and $\text {Z} \text {Z}$). These samples are generated using NLO NNPDF 3.0 for the matrix element calculation. The $\gamma \gamma$ background process leading to two charged leptons in the final state, $\gamma \gamma \rightarrow \ell ^+ \ell ^-$, is simulated using LPair [68, 69] interfaced with pythia 6 and using the default $\gamma$-PDF of Suri–Yennie [70]. This contribution is split into three components, since the interaction at each proton vertex process can be elastic or inelastic.

The total cross sections of $\text {W} \text {Z}$ and $\text {Z} \text {Z}$ diboson samples are normalized to the NLO prediction calculated with mcfm  v6.6 [71], whereas the cross sections of the $\text {W} \text {W}$ samples are normalized to the NNLO prediction [72]. The total cross section of the ${\text {t}} {\bar{\text {t}}}$ production is normalized to the prediction with NNLO accuracy in QCD and next-to-next-to-leading logarithmic (NNLL) accuracy in soft gluon resummation calculated with Top++  2.0 [73]. The single top and $\gamma \gamma$ background distributions are normalized to the cross sections calculated by their respective event generators.

It is possible for hadrons to mimic the signature of an electron in the detector. The main processes that contribute to this background are W + jet production, when the W decays leptonically, and QCD multijet events. Such backgrounds are nonnegligible only in the electron channel.

The contamination of the signal region by events containing hadrons misidentified as electrons is estimated using a control region where two electrons of the same sign are required. This control region mainly contains events with hadrons misidentified as electrons and events originating from the DY process when the charge of one electron is incorrectly attributed. The probability of charge misidentification is obtained as a function of $p_{\textrm{T}}$ and $\eta$ of each electron in the Z peak region ($81< m_{\ell \ell } <101\,\text {Ge\hspace{-.08em}V}$), where the hadron contamination is negligible even in the control region. These probabilities are then used to estimate the charge misidentification rate for other values of $m_{\ell \ell }$. The difference between the observed number of events in the control region and the estimated charge misidentification rate is assumed to be the contamination from hadron background. We observe that the numbers of misidentified-lepton events in the same-sign electron sample and in the signal (opposite-sign electron) sample are compatible.

**Fig. 1:**
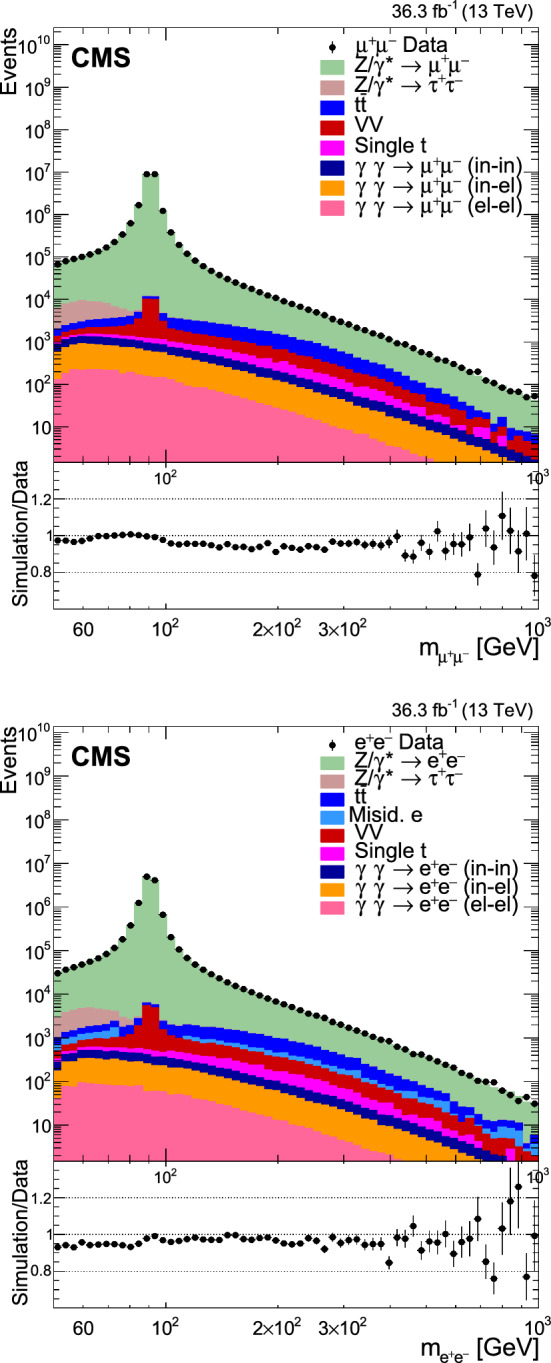
Distributions of events passing the selection requirements in the muon (left) and electron channels (right). Each plot also presents in the lower part a ratio of simulation over data. Only statistical uncertainties are shown as error bars on the data points, whereas the ratio presents the statistical uncertainty in the simulation and the data. The plots show the number of events without normalization to the bin width. The different background contributions are discussed in the text

**Fig. 2:**
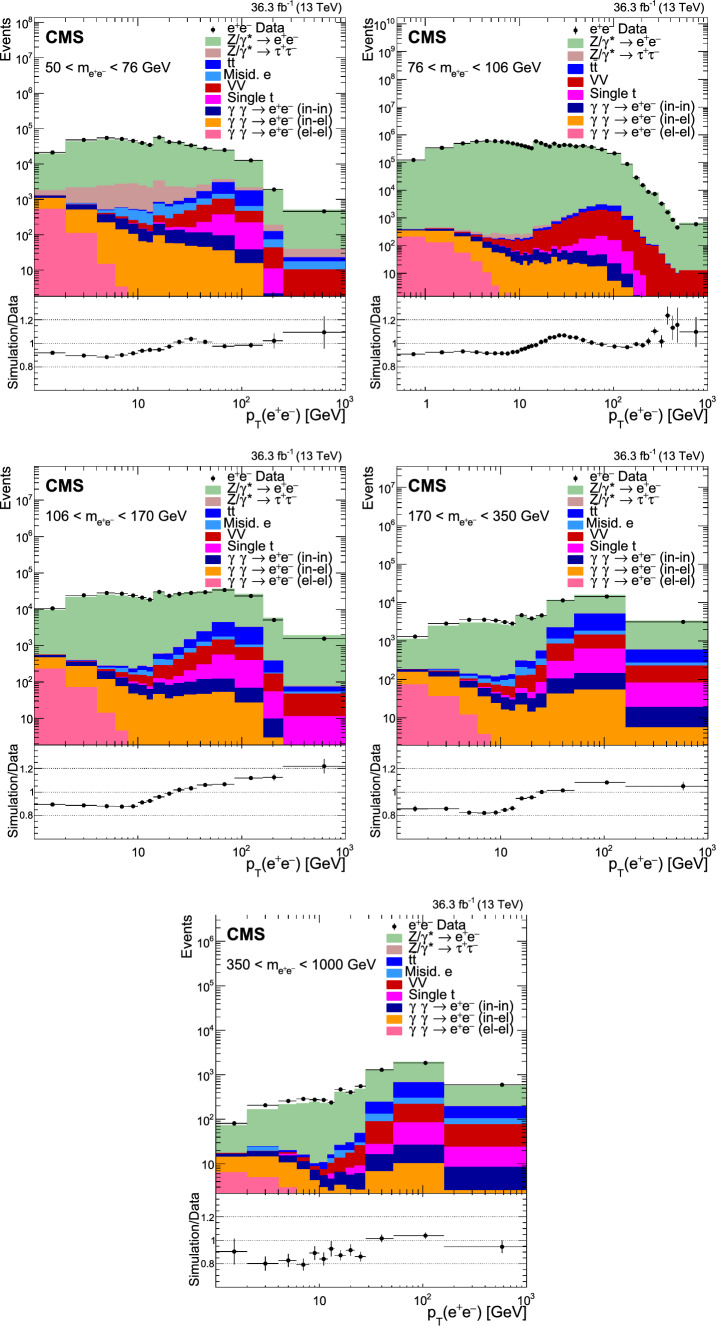
Distributions of events passing the selection requirements in the electron channel as a function of the dilepton $p_{\textrm{T}}$ in five ranges of invariant mass: 50–76$\,\text {Ge\hspace{-.08em}V}$ (upper left), 76–106$\,\text {Ge\hspace{-.08em}V}$ (upper right), 106–170$\,\text {Ge\hspace{-.08em}V}$ (middle left), 170–350 $\,\text {Ge\hspace{-.08em}V}$ (middle right), and 350–1000$\,\text {Ge\hspace{-.08em}V}$ (lower). More details are given in Fig. 1

The number of events at the reconstructed level is compared with the sum of the contributions from signal and backgrounds. In Fig. 1, the dilepton mass spectrum is shown for both the electron and and the muon channels, whereas Fig. 2 shows the $p_{\textrm{T}} (\ell \ell )$ distributions in various $m_{\ell \ell }$ bins for the electron channel only. Globally, the background contamination is lower than 1%. The background becomes around 10% for $m_{\ell \ell }$ outside of the Z boson mass peak and up to 30% in some bins. The simulated samples are processed through a Geant4 [74] based simulation of the CMS detector, with the same reconstruction algorithms as of data. They also include a pileup profile that is reweighed to match the profile of the data.

## Measured observables and unfolding procedure

The measurement of the DY cross section is carried out with respect to the $p_{\textrm{T}}$ and $\varphi ^{*}_{\eta }$ of the dilepton pairs produced inclusively, and with respect to $p_{\textrm{T}}$ for pairs produced in association with at least one jet. For the inclusive case, the measurement is divided into five invariant mass bins: 50–76, 76–106, 106–170, 170–350, 350–1000$\,\text {Ge\hspace{-.08em}V}$; the last bin is not included when requiring at least one jet because of the small number of events available. The measurement of the ratio of cross section in mass bins 50–76, 106–170, 170–350, 350–1000$\,\text {Ge\hspace{-.08em}V}$ to the cross section around the Z mass peak(76–106$\,\text {Ge\hspace{-.08em}V}$) is also performed. The bin widths are chosen to be as small as possible, based on the detector resolution and the number of events.

To correct for the detector resolution and the efficiency of the selection, an unfolding procedure is applied to the measured distributions one dimensionally in each mass bin. To obtain the particle-level distributions from the reconstructed distributions, the unfolding uses a response matrix based on the simulated signal sample. To unfold, the D’Agostini iterative method with early stopping is used as implemented in RooUnfold [75]. The result, converging towards the maximum likelihood estimate, is affected by fluctuations increasing with the number of iterations. The fluctuations are studied using pseudo-experiments for each number of iterations following the method used in Ref. [76]. The procedure is stopped just before the fluctuations become significant with respect to the statistical uncertainty. The number of iterations ranges between 4 and 25.

The particle level refers to stable particles ($c\tau >1\,{\textrm{cm}}$), other than neutrinos, in the final state. To correct for energy losses due to QED FSR, leptons are “dressed”, i.e., all the prompt photons with a distance smaller than $\varDelta R=0.1$ to the lepton axis are added to the lepton momentum. The cross section is extracted in the following phase space: leading and subleading dressed leptons satisfying $p_{\textrm{T}} > 25$ and 20$\,\text {Ge\hspace{-.08em}V}$ and $|\eta |<2.4$. When at least one additional jet is required, it must satisfy $p_{\textrm{T}} >30\,\text {Ge\hspace{-.08em}V}$, $|y |<2.4$, and be spatially separated from the dressed leptons by $\varDelta R>0.4$.

The cross sections are first extracted separately for the electron and muon channels. They are compatible for all studied distributions and the two channels are combined to reduce the statistical uncertainties. The combined differential cross sections are calculated bin-by-bin as the weighted mean values of the differential cross sections of the two channels. The systematic and statistical uncertainties are obtained using the linear combination method described in Ref. [77], considering as fully correlated the uncertainties in the jet energy scale and resolution, the pileup, the background subtraction, b tagging, and the integrated luminosity. Other uncertainties are considered as uncorrelated.

## Systematic uncertainties

Several sources of uncertainties in the measurement are considered. The integrated luminosity is measured with a precision of $1.2\%$ [78], which results in a relative uncertainty of almost the same value in the measurement. Small variations are caused by the subtraction of the background contributions estimated from the simulation.

The uncertainties coming from the lepton trigger efficiencies are estimated by varying the applied scale factors up and down by one standard deviation. The uncertainties from identification and reconstruction efficiencies are estimated for various sources including QED FSR, resolution, background modeling, and the tag object selection in the tag-and-probe procedure, as well as the statistical component treated separately for each scale factor in $p_{\textrm{T}}$ and $\eta$ of the lepton [26]. The efficiency uncertainties include a one percent effect in the L1 trigger caused by a timing problem in ECAL endcaps. The lepton energy scale uncertainties are estimated by varying the lepton energy and $p_{\textrm{T}}$ by $\pm 1$ standard deviation (reach 0.75% (0.5%) for electrons (muons) depending on $\eta$ and $p_{\textrm{T}}$). Uncertainties coming from the lepton energy resolution are estimated by spreading the lepton energy using the generator-level information.

The uncertainty in the jet energy scale is estimated by varying the jet momenta in data by 2.5–5%, depending on the energy and pseudorapidity of the jet. The uncertainty in the jet energy resolution is estimated by varying the smearing factor used to match the simulated jet energy resolution to data by ±1 standard deviation around its central value.

A systematic uncertainty is attributed to the normalisation of the background samples estimated by Monte Carlo event generators. The theoretical uncertainty in the cross section of the dominant ${{\text {t}} {}{\bar{\text {t}}}}$ background is $\approx$6%, using the Top++  2.0 program and including scale and PDF variations. The uncertainties in the other background cross sections are smaller. In particular, it has been verified that 6% covers the differences of the $\gamma \gamma \rightarrow \ell ^+ \ell ^-$samples generated using Suri–Yennie and LuxQED [79, 80] photon PDFs. In a conservative way, the uncertainties in all other Monte Carlo based background estimates are also estimated to be 6%. This uncertainty is applied to fully-elastic, semi- and fully-inelastic cases.

**Fig. 3:**
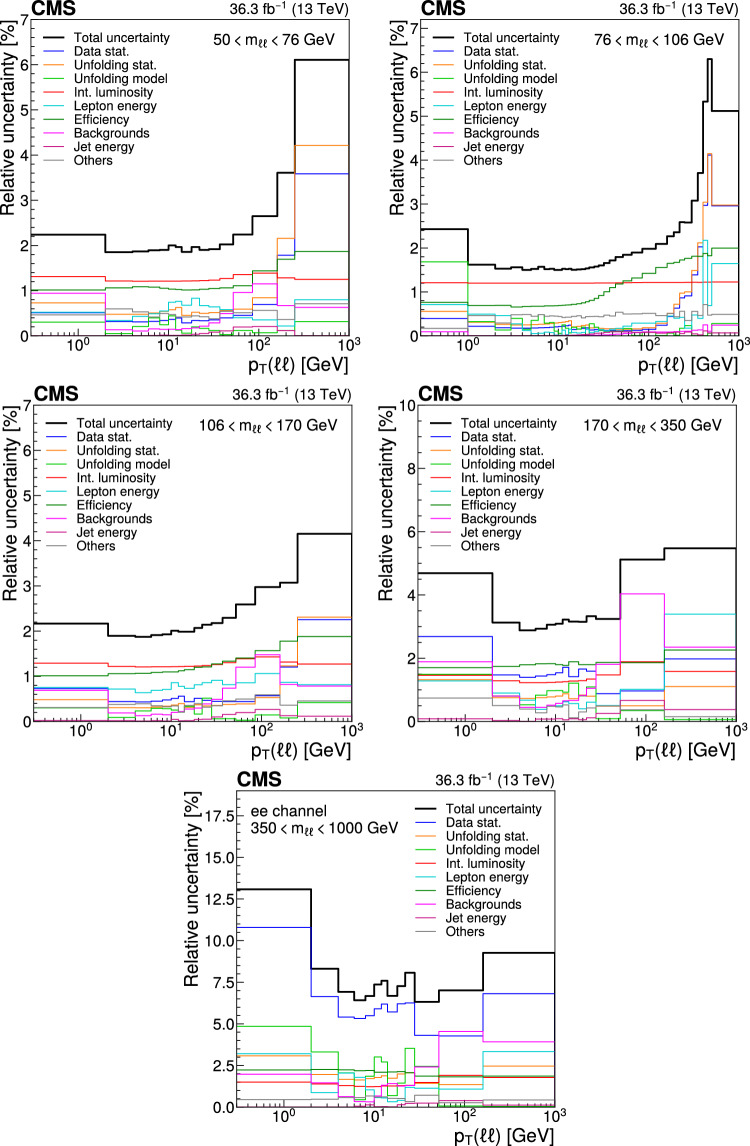
Estimates of the uncertainties in inclusive differential cross sections in $p_{\textrm{T}} (\ell \ell )$ in various invariant mass ranges: $50< m_{\ell \ell } <76\,\text {Ge\hspace{-.08em}V}$ (upper left), $76< m_{\ell \ell } <106\,\text {Ge\hspace{-.08em}V}$ (upper right), $106< m_{\ell \ell } <170\,\text {Ge\hspace{-.08em}V}$ (middle left), $170< m_{\ell \ell } <350\,\text {Ge\hspace{-.08em}V}$ (middle right), and $350< m_{\ell \ell } <1000\,\text {Ge\hspace{-.08em}V}$ (lower). The black line is the quadratic sum of the colored lines

The uncertainty in the misidentified electron background estimation using same-sign events is obtained using an uncertainty in the charge misidentification estimation of about 10% per electron at $p_{\textrm{T}} (\text {e})=150\,\text {Ge\hspace{-.08em}V}$, rising with $p_{\textrm{T}} (\text {e})$. A 20% total uncertainty in the charge misidentification is used and propagated to the estimate of this background.

Alternative pileup profiles are generated by varying the amount of pileup events by 5%, and the difference to the nominal sample is propagated to the final results.

**Fig. 4:**
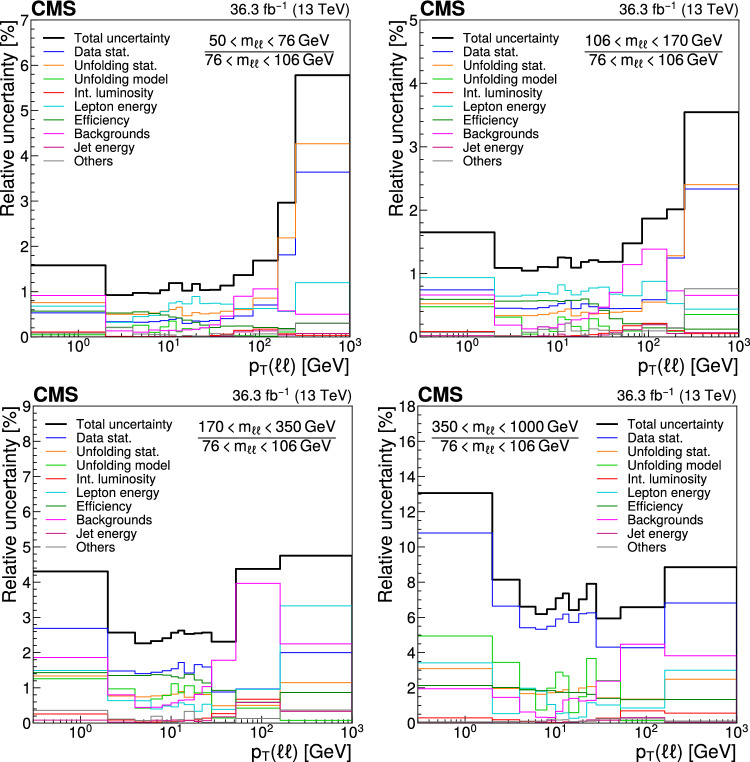
Estimates of the uncertainties in inclusive differential cross section ratios in $p_{\textrm{T}} (\ell \ell )$ for invariant mass ranges with respect to the peak region $76< m_{\ell \ell } <106\,\text {Ge\hspace{-.08em}V}$: $50< m_{\ell \ell } <76\,\text {Ge\hspace{-.08em}V}$ (upper left), $106< m_{\ell \ell } <170\,\text {Ge\hspace{-.08em}V}$ (upper right), $170< m_{\ell \ell } <350\,\text {Ge\hspace{-.08em}V}$ (lower left), and $350< m_{\ell \ell } <1000\,\text {Ge\hspace{-.08em}V}$ (lower right). The black line is the quadratic sum of the colored lines

The unfolding model uncertainty is estimated by reweighting the simulated sample to match the data shape for each distribution, and using this as an alternate model for unfolding. The difference with respect to the results obtained with the simulated sample is assigned as the uncertainty. The statistical uncertainty coming from the limited sample size is also included, provided by the RooUnfold package.

The systematic uncertainties are propagated to the measurement through the unfolding procedure by computing new response matrices varying the quantities by one standard deviation up and down. All the experimental uncertainties are symmetrized by taking the average of the deviations from the central value. The uncertainty sources are independent and the resulting uncertainties are added in quadrature.

For the inclusive measurement the main sources of uncertainties are the integrated luminosity measurement, the identification and trigger efficiency corrections of the leptons, and the energy scale of the leptons. For the DY + $\ge$1 jet case, the major uncertainties come from the jet energy scale and the unfolding model. The estimates of systematic uncertainties for the inclusive differential cross sections in $p_{\textrm{T}} (\ell \ell )$ for various $m_{\ell \ell }$ ranges are shown in Fig. 3.

When calculating the cross section ratios, for each $p_{\textrm{T}} (\ell \ell )$ or $\varphi ^{*}_{\eta }$ bin, all uncertainties are taken as fully correlated between the numerator and the denominator, except the data and Monte Carlo statistical uncertainties. The total uncertainty corresponds to the quadratic sum of the sources. The estimates of systematic uncertainties for the ratios of the inclusive $p_{\textrm{T}} (\ell \ell )$ distributions are shown in Fig. 4.

## Theory predictions

The measured data are compared with the mg5_amc + pythia 8 baseline sample described in Sect. 4. The QCD scale uncertainties are estimated by varying the renormalisation and factorisation scales simultaneously by factors of 2 and 1/2 (omitting the variations in opposite direction and taking the envelope). The strong coupling ($\alpha _\textrm{S}$) and PDF uncertainties are estimated as the standard deviation of weights from the replicas provided in the NNPDF 3.0 PDF set [62].

An event sample at NNLO with a jet merging method is generated with $\textsc {MiNNLO}_\textrm{PS}$ [81, 82]. The coupling $\alpha _\textrm{S}$ is evaluated independently at each vertex at a scale that depends on the kinematic configuration. Sudakov form factors are used to interpolate between the scales. The NNLO version of the NNPDF 3.1 PDF set [83] is used along with the pythia version 8 [60] for the parton showers based on the CP5 tune [84] and multiparton interactions (MPI), but including a harder primordial $k_{\textrm{T}}$ of 2.2 $\,\text {Ge\hspace{-.08em}V}$ obtained from tuning the $k_{\textrm{T}}$ parameter to describe the observed $\varphi ^{*}_{\eta }$ distribution of Ref. [26].

The results are also compared with a third prediction from the parton branching (PB) TMD method [14, 15] obtained from Cascade 3 [85]. This prediction is of particular interest since the initial-state parton showers are fully determined by TMD and their backward PB evolution, and therefore are free of tuning parameters. The matrix element calculation is performed at NLO for $\text {Z} + 0$ jet using mg5_amc for the inclusive distributions (labelled mg5_amc (0 jet at NLO)+ PB (Cascade)), and for $\text {Z} + 1$ jet for the distributions where one jet is required in the final state (labelled mg5_amc (1 jet at NLO)+ PB (Cascade)). Initial-state parton showers, provided by the PB TMD method are matched to the NLO matrix element [86], using the latest TMD PB set: PB-NLO-HERAI+II-2018-set2 [87]. The final parton shower, hadronization, and QED FSR steps are performed with pythia 6 [88]. This approach is equivalent to the inclusion of the next-to-leading logarithmic soft-gluon resummation on top of the fixed-order NLO calculations. The theoretical uncertainties in the cross section are estimated by variation of scales and from TMD uncertainties. This approach is expected to describe the inclusive cross section at low $p_{\textrm{T}} (\ell \ell )$ ($< 20\,\text {Ge\hspace{-.08em}V}$) well, and to fail for larger $p_{\textrm{T}} (\ell \ell )$, since higher-order matrix element contributions are missing, as already observed for the Z boson mass peak range [26]. Recently, this approach has been developed to include multi-jet merging [89] at LO, which allows a larger $p_{\textrm{T}} (\ell \ell )$ region to be described as well.

A fourth prediction is based on an independent approach relying on TMDs obtained from fits to DY and Z boson measurements at different energies [90, 91] using an NNLO evolution. The corresponding numerical evaluations are provided by the arTeMiDe 2.02 code [92]. The resummation corresponds to an $\hbox {N}^3$LL approximation. The uncertainty is obtained from scale variations. Due to the approximation of ordering among the scales, the prediction has a limited range of validity for the calculation of: $p_{\textrm{T}} (\ell \ell ) < 0.2 \ m_{\ell \ell }$. Predictions for the $\varphi ^{*}_{\eta }$ cross section dependence as well as the 1 jet case are not provided by arTeMiDe. The arTeMiDe sample does not include the QED FSR; a correction is derived from the pythia 8 shower in the mg5_amc sample. The uncertainty is derived by taking the difference with respect to corrections derived from the powheg sample described in Ref. [26]. This uncertainty is smaller than 1% for $p_{\textrm{T}} (\ell \ell ) <0.2\ m_{\ell \ell }$

Two more predictions are obtained from the Geneva 1.0-RC3 program [93–95] combining higher-order resummation with a DY calculation at NNLO. Originally, the resummation was carried out at NNLL including partially $\hbox {N}^{3}$LL on the 0-jettiness variable $\tau _0$ [96]. More recently it includes the $q_\textrm{T}$ resummation at $\mathrm {N^3LL}$ in the Radish formalism [97, 98] for the 0 jet case, whereas it keeps the 1-jettiness resummation for the 1 jet case. Two samples are generated, one in the 0-jettiness approach and one in the $q_\textrm{T}$ resummation approach. The calculation uses the PDF4LHC15 NNLO [99] PDF set with $\alpha _\textrm{S} (m_{\text {Z}})=0.118$, the world average. The events are showered using a specially modified version of pythia 8, which is also used for nonperturbative effects and QED radiation in the initial and final states using a modified tune based on CUETP8M1. The theoretical uncertainties are estimated by variation of scales and from the resummation as described in Ref. [94]. No uncertainty is assigned to the jetiness resummation.

## Results and discussion

### $p_{\textrm{T}} (\ell \ell )$ results

**Fig. 5:**
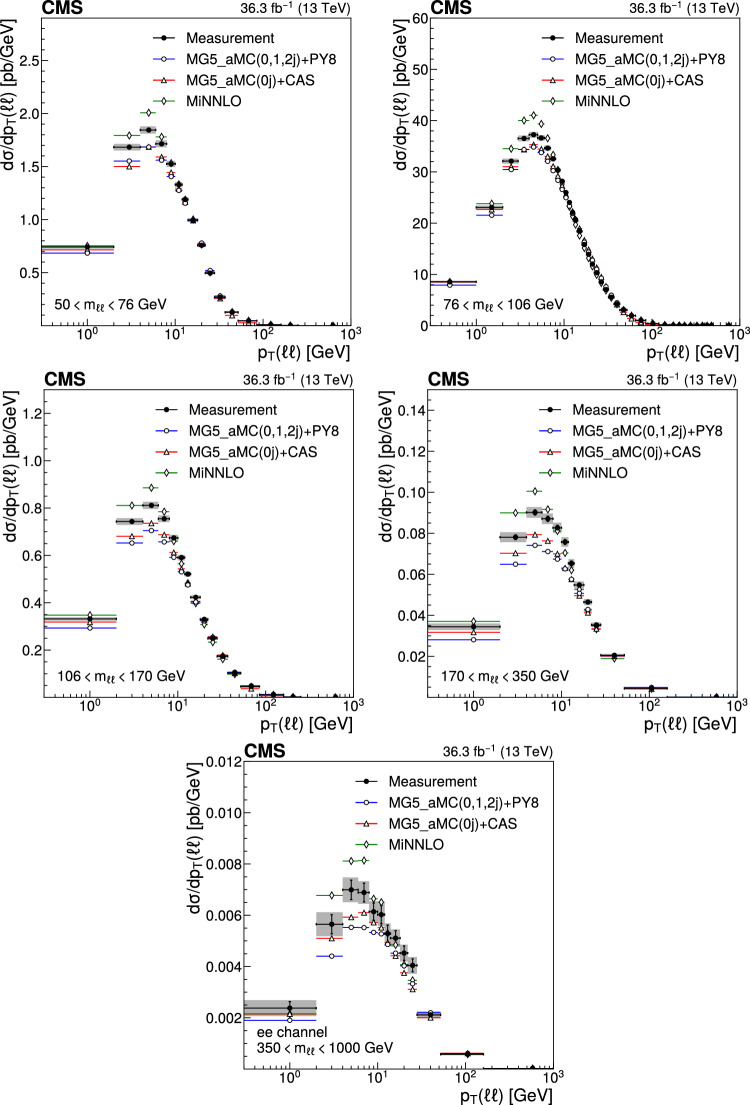
Differential cross sections in $p_{\textrm{T}} (\ell \ell )$ in various invariant mass ranges: $50< m_{\ell \ell } <76\,\text {Ge\hspace{-.08em}V}$ (upper left), $76< m_{\ell \ell } <106\,\text {Ge\hspace{-.08em}V}$ (upper right), $106< m_{\ell \ell } <170\,\text {Ge\hspace{-.08em}V}$ (middle left), $170< m_{\ell \ell } <350\,\text {Ge\hspace{-.08em}V}$ (middle right), and $350< m_{\ell \ell } <1000\,\text {Ge\hspace{-.08em}V}$ (lower). The error bars on data points (black dots) correspond to the statistical uncertainty of the measurement and the shaded bands around the data points correspond to the total experimental uncertainty. The measurement is compared with mg5_amc (0, 1, and 2 jets at NLO) + pythia 8 (blue dots), $\textsc {MiNNLO}_\textrm{PS}$ (green diamonds) and mg5_amc (0 jet at NLO)+ PB (Cascade) (red triangles)

**Fig. 6:**
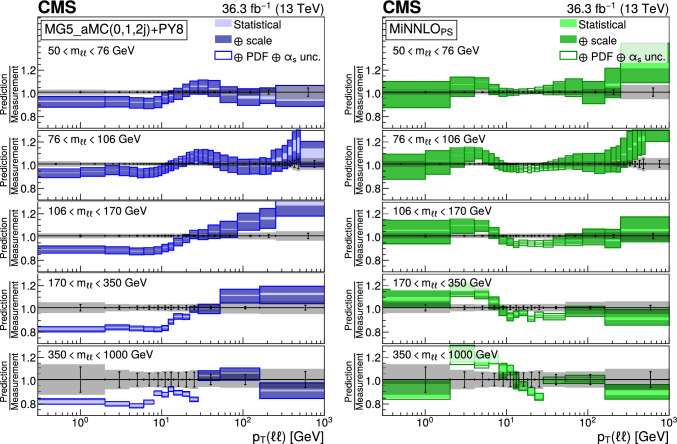
Comparison to Monte Carlo predictions based on a matrix element with parton shower merging. The ratio of mg5_amc (0, 1, and 2 jets at NLO) + pythia 8 (left) and $\textsc {MiNNLO}_\textrm{PS}$ (right) predictions to the measured differential cross sections in $p_{\textrm{T}} (\ell \ell )$ are presented for various $m_{\ell \ell }$ ranges. The error bars correspond to the statistical uncertainty of the measurement and the shaded bands to the total experimental uncertainty. The light color band corresponds to the statistical uncertainty of the simulation and the dark color band includes the scale uncertainty. The largest bands include PDF and $\alpha _\textrm{S}$ uncertainties, added in quadrature

**Fig. 7:**
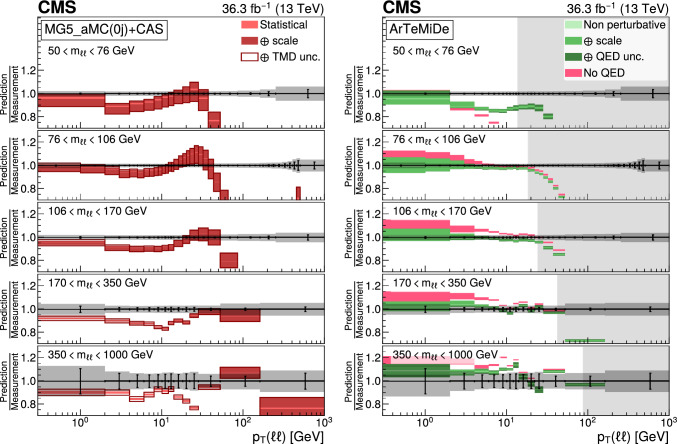
Comparison to TMD based predictions. The ratio of mg5_amc (0 jet at NLO) + PB (Cascade) (left) and arTeMiDe (right) predictions to the measured differential cross sections in $p_{\textrm{T}} (\ell \ell )$ are presented for various $m_{\ell \ell }$ ranges. The error bars correspond to the statistical uncertainty of the measurement and the shaded bands to the total experimental uncertainty. The light (dark) green band around arTeMiDe predictions represent the nonperturbative (QCD scale) uncertainties, the darker green representing the QED FSR correction uncertainties. The range of invalidity is shaded with a gray band. The light color band around Cascade prediction corresponds to the statistical uncertainty of the simulation and the dark color band includes the scale uncertainty. The largest bands include TMD uncertainty, added in quadrature

**Fig. 8:**
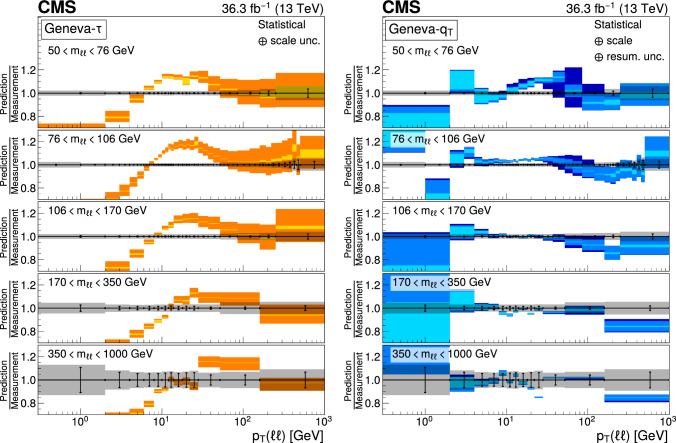
Comparison to resummation based predictions. The ratio of Geneva-$\tau$ (left) and Geneva-$q_\textrm{T}$ (right) predictions to the measured differential cross sections in $p_{\textrm{T}} (\ell \ell )$ are presented for various $m_{\ell \ell }$ ranges. The error bars correspond to the statistical uncertainty of the measurement and the shaded bands to the total experimental uncertainty. The light color bands around the predictions represents the statistical uncertainties and the middle color bands represents the scale uncertainties. The dark outer bands of Geneva-$q_\textrm{T}$ prediction represent the resummation uncertainties

**Fig. 9:**
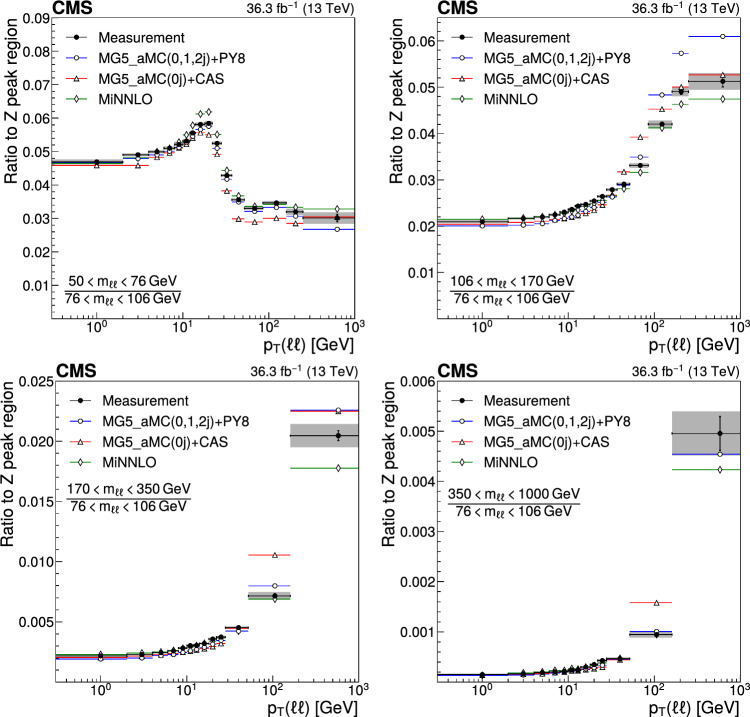
Ratios of differential cross sections in $p_{\textrm{T}} (\ell \ell )$ for invariant mass ranges with respect to the peak region $76< m_{\ell \ell } <106\,\text {Ge\hspace{-.08em}V}$: $50< m_{\ell \ell } <76\,\text {Ge\hspace{-.08em}V}$ (upper left), $106< m_{\ell \ell } <170\,\text {Ge\hspace{-.08em}V}$ (upper right), $170< m_{\ell \ell } <350\,\text {Ge\hspace{-.08em}V}$ (lower left), and $350< m_{\ell \ell } <1000\,\text {Ge\hspace{-.08em}V}$ (lower right). Details on the presentation of the results are given in Fig. 5 caption

**Fig. 10:**
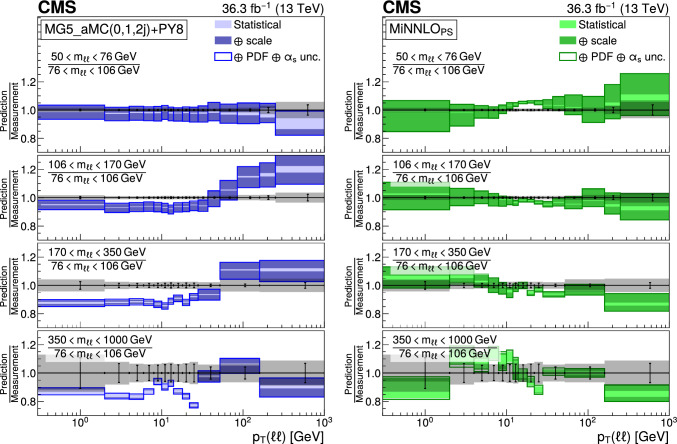
Comparison to Monte Carlo predictions based on a matrix element with parton shower merging. The distributions show the ratio of differential cross sections as a function of $p_{\textrm{T}} (\ell \ell )$ for a given $m_{\ell \ell }$ range to the cross section at the peak region $76< m_{\ell \ell } <106\,\text {Ge\hspace{-.08em}V}$. The predictions are mg5_amc (0, 1, and 2 jets at NLO) + pythia 8 (left) and $\textsc {MiNNLO}_\textrm{PS}$ (right). Details on the presentation of the results are given in Fig. 6 caption

**Fig. 11:**
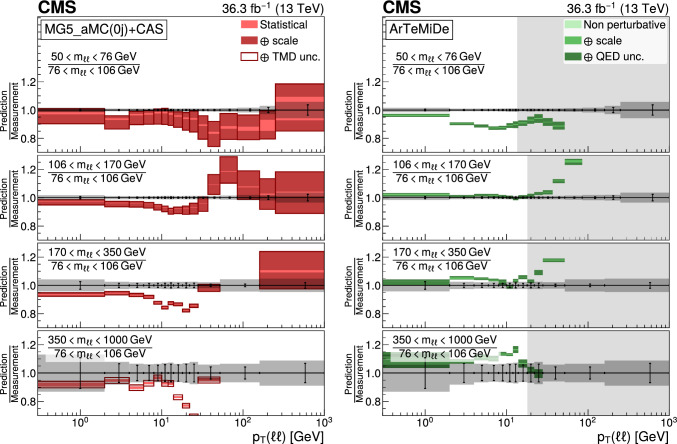
Comparison to TMD based predictions. The distributions show the ratio of differential cross sections as a function of $p_{\textrm{T}} (\ell \ell )$ for a given $m_{\ell \ell }$ range to the cross section at the peak region $76< m_{\ell \ell } <106\,\text {Ge\hspace{-.08em}V}$. The predictions are mg5_amc (0 jet at NLO) + PB (Cascade) (left) and arTeMiDe (right). Details on the presentation of the results are given in Fig. 7 caption

**Fig. 12:**
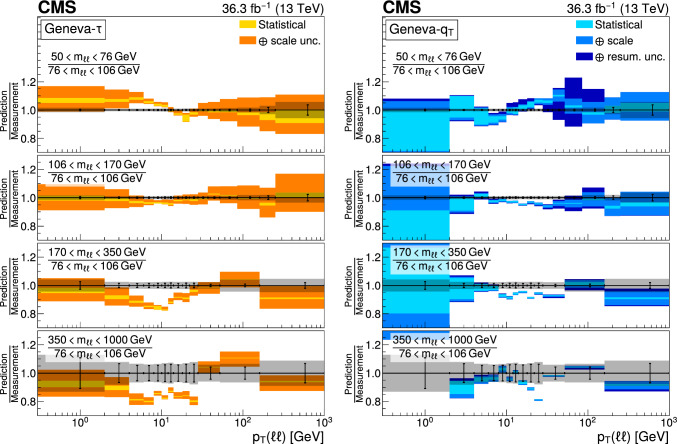
Comparison to resummation based predictions. The distributions show the ratio of differential cross sections as a function of $p_{\textrm{T}} (\ell \ell )$ for a given $m_{\ell \ell }$ range to the cross section at the peak region $76< m_{\ell \ell } <106\,\text {Ge\hspace{-.08em}V}$. The predictions are Geneva-$\tau$ (left) and Geneva-$q_\textrm{T}$ (right). Details on the presentation of the results are given in Fig. 8 caption

The differential cross sections in $p_{\textrm{T}} (\ell \ell )$ are shown in Fig. 5 for invariant mass ranges between 50$\,\text {Ge\hspace{-.08em}V}$ and 1$\,\text {Te\hspace{-.08em}V}$. Because of the lack of precision of the muon transverse momentum measurement at high $p_{\textrm{T}}$, the cross section measurement in the highest mass range is based on the electron channel only. The ratio of the predictions to the data are presented in Figs. 6, 7 and 8. The comparison with different predictions is discussed later in the text. The ratios of the unfolded distributions for invariant masses outside the Z boson peak to the distribution within the Z boson peak ($76< m_{\ell \ell } <106\,\text {Ge\hspace{-.08em}V}$) are shown in Fig. 9, and the comparisons to predictions in Figs. 10, 11 and 12.

**Fig. 13:**
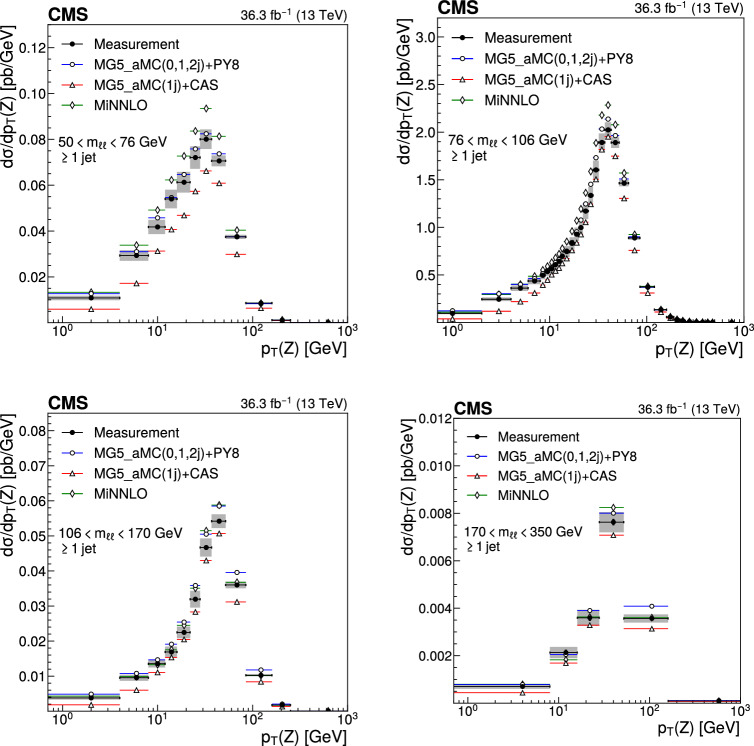
Differential cross sections in $p_{\textrm{T}} (\ell \ell )$ for one or more jets in various invariant mass ranges: $50< m_{\ell \ell } <76\,\text {Ge\hspace{-.08em}V}$ (upper left), $76< m_{\ell \ell } <106\,\text {Ge\hspace{-.08em}V}$ (upper right), $106< m_{\ell \ell } <170\,\text {Ge\hspace{-.08em}V}$ (lower left), and $170< m_{\ell \ell } <350\,\text {Ge\hspace{-.08em}V}$ (lower right). The measurement is compared with mg5_amc (0, 1, and 2 jets at NLO) + pythia 8 (blue dots), $\textsc {MiNNLO}_\textrm{PS}$ (green diamonds) and mg5_amc (1 jet at NLO)+ PB (Cascade) (red triangles). Details on the presentation of the results are given in Fig. 5 caption

**Fig. 14:**
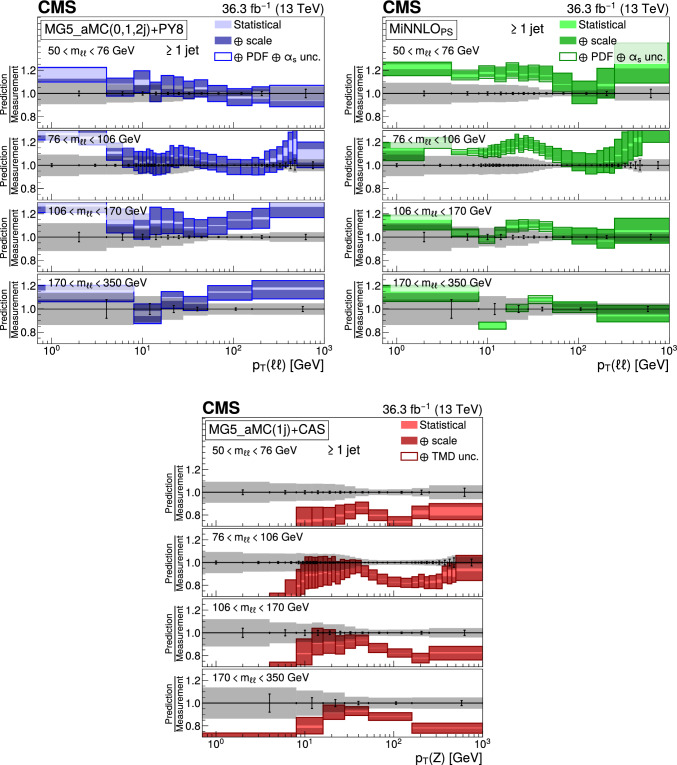
Comparison of the differential cross sections in $p_{\textrm{T}} (\ell \ell )$ to predictions in various invariant mass ranges for the one or more jets case. The measurement is compared with mg5_amc (0, 1, and 2 jets at NLO) + pythia 8 (upper left), $\textsc {MiNNLO}_\textrm{PS}$ (upper right) and mg5_amc (1 jet at NLO) + PB (Cascade) (lower). Details on the presentation of the results are given in Fig. 6 caption

**Fig. 15:**
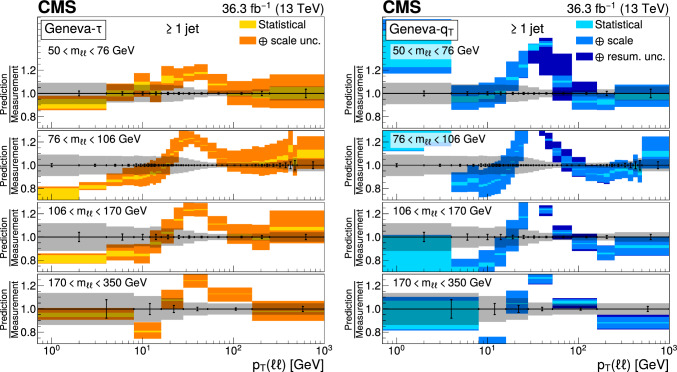
Comparison of the differential cross sections in $p_{\textrm{T}} (\ell \ell )$ to predictions in various invariant mass ranges for the one or more jets case. The measurement is compared with Geneva-$\tau$ (left) and Geneva-$q_\textrm{T}$ (right) predictions. Details on the presentation of the results are given in Fig. 8 caption

**Fig. 16:**
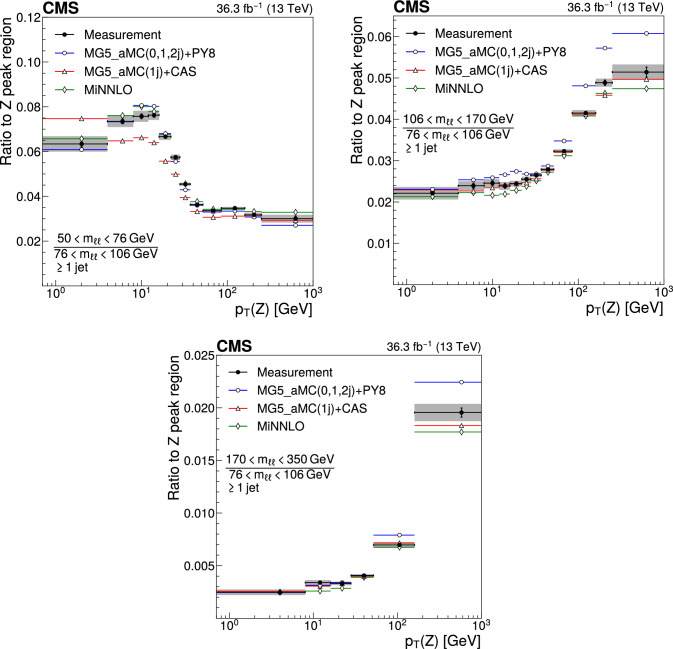
Ratios of differential cross sections in $p_{\textrm{T}} (\ell \ell )$ for one or more jets in various invariant mass ranges with respect to the peak region $76< m_{\ell \ell } <106\,\text {Ge\hspace{-.08em}V}$: $50< m_{\ell \ell } <76\,\text {Ge\hspace{-.08em}V}$ (upper left), $106< m_{\ell \ell } <170\,\text {Ge\hspace{-.08em}V}$ (upper right), and $170< m_{\ell \ell } <350\,\text {Ge\hspace{-.08em}V}$ (lower). The measurement is compared with mg5_amc (0, 1, and 2 jets at NLO) + pythia 8 (blue dots), $\textsc {MiNNLO}_\textrm{PS}$ (green diamonds) and mg5_amc (1 jet at NLO)+ PB (Cascade) (red triangles). Details on the presentation of the results are given in Fig. 5 caption

**Fig. 17:**
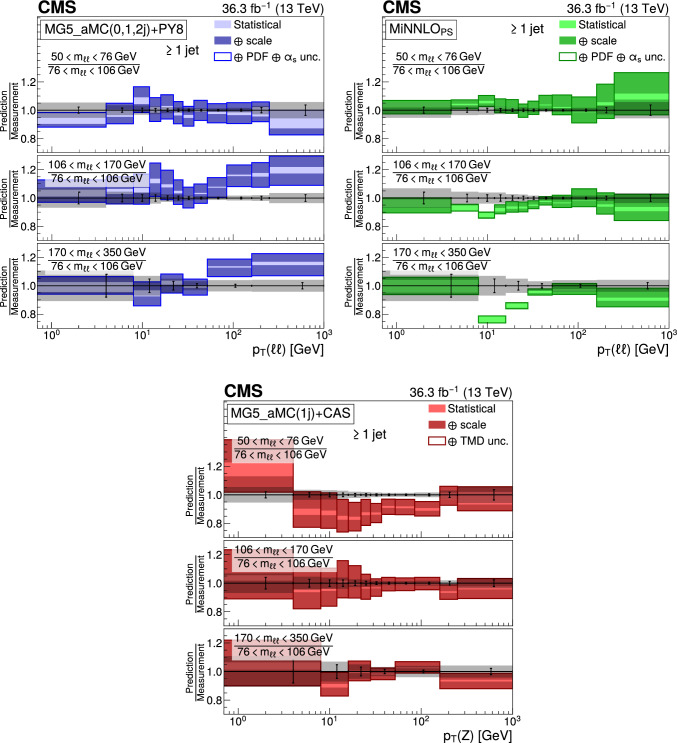
Comparison of the ratios of differential cross sections in $p_{\textrm{T}} (\ell \ell )$ for one or more jets in various invariant mass ranges with respect to the peak region $76< m_{\ell \ell } <106\,\text {Ge\hspace{-.08em}V}$. The measured ratio is compared with mg5_amc (0, 1, and 2 jets at NLO) + pythia 8 (upper left), $\textsc {MiNNLO}_\textrm{PS}$ (upper right) and mg5_amc (1 jet at NLO) + PB (Cascade) (lower). Details on the presentation of the results are given in Fig. 6 caption

**Fig. 18:**
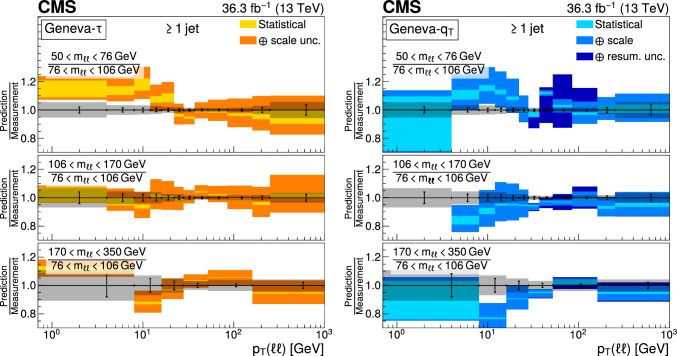
Comparison of the ratios of differential cross sections in $p_{\textrm{T}} (\ell \ell )$ for one or more jets in various invariant mass ranges with respect to the peak region $76< m_{\ell \ell } <106\,\text {Ge\hspace{-.08em}V}$. The measured ratio is compared with Geneva-$\tau$ (left) and Geneva-$q_\textrm{T}$ (right) predictions. Details on the presentation of the results are given in Fig. 8 caption

The measured cross sections are presented in Fig. 13 as a function of $p_{\textrm{T}} (\ell \ell )$ for at least one jet, for the same mass ranges except the highest. Ratios of the predictions to the data are presented in Figs. 14 and 15. The ratio of these differential cross sections for various mass ranges with respect to the same distribution in the Z boson peak region are shown in Fig. 16, and the comparisons to predictions in Figs. 17 and 18.

The measurements show that the differential cross sections in $p_{\textrm{T}} (\ell \ell )$ are rising from small $p_{\textrm{T}} (\ell \ell )$ values up to a maximum between 4 and 6$\,\text {Ge\hspace{-.08em}V}$ and then falling towards large $p_{\textrm{T}} (\ell \ell )$ (Fig. 5). For these cross sections, the variation of the dilepton invariant mass does not have a visible effect on the peak position (around 5$\,\text {Ge\hspace{-.08em}V}$) or on the rising shape for the values below the peak. However, the increase of $m_{\ell \ell }$ results in a broader distribution for $p_{\textrm{T}} (\ell \ell )$ values above the peak. These effects are highlighted by the cross section ratios presented in Fig. 9. It has to be noted that the rising ratio for the lowest $m_{\ell \ell }$ range (Fig. 9 top left) up to a $p_{\textrm{T}} (\ell \ell )$ value of 20$\,\text {Ge\hspace{-.08em}V}$ is due to QED radiative effects on the final-state leptons (photon radiations at $\varDelta R(\ell ,\gamma )>0.1$) inducing migrations from the Z mass peak towards lower masses. When a jet with a large transverse momentum is required (Fig. 13), the peak is shifted towards larger $p_{\textrm{T}} (\ell \ell )$ values corresponding to the jet selection threshold, here 30$\,\text {Ge\hspace{-.08em}V}$ regardless of the $m_{\ell \ell }$. As in the inclusive case, the distributions become broader for $p_{\textrm{T}} (\ell \ell )$ values larger than the peak for increasing $m_{\ell \ell }$.

A description of these measurements based on QCD requires both multi-gluon resummation and a fixed-order matrix element. The description of the distributions at small $p_{\textrm{T}} (\ell \ell )$ values requires an approach taking into account initial-state nonperturbative and perturbative multi-gluon resummation. The falling behaviour at large $p_{\textrm{T}} (\ell \ell )$ is sensitive to hard QCD radiation, which is expected to be well described by matrix element calculations including at least NLO corrections. The size of the QCD radiation is driven by the available kinematic phase space and the value of $\alpha _\textrm{S}$. An increase of $m_{\ell \ell }$ extends the phase space for hard radiations, slightly compensated by the decrease of $\alpha _\textrm{S}$ with increasing $m_{\ell \ell }$. The tail at large $p_{\textrm{T}} (\ell \ell )$ is dominated by jet multiplicities above one. For the inclusive cross sections, the resummation effects are concentrated at small $p_{\textrm{T}} (\ell \ell )$. The value of the maximum of the distributions is expected to depend weakly on $m_{\ell \ell }$. In the presence of a hard jet, multiple gluon emissions also affect the perturbative region located in $\eta$ between the jet and the vector boson. The corresponding cross section measurements therefore provide additional constraints on the resummation treatment in the predictions.

The mg5_amc + pythia 8 prediction describes the data well globally (Fig. 6), although it predicts a too-small cross section for $p_{\textrm{T}} (\ell \ell )$ values below 30$\,\text {Ge\hspace{-.08em}V}$ in the inclusive case. This disagreement is more pronounced at higher $m_{\ell \ell }$ and reaches about 20% for masses above 170$\,\text {Ge\hspace{-.08em}V}$. The low-$p_{\textrm{T}} (\ell \ell )$ region is sensitive to gluon resummation. In mg5_amc, the resummation effects are simulated by the parton shower, modelled in pythia 8 depending on parameters tuned on previously published measurements, including DY cross sections in the Z boson mass peak region. It has to be noted that the low $p_{\textrm{T}} (\ell \ell )$ spectrum is sensitive to the choice of the tuned parameters [84] and that no related systematic uncertainty is available. The large $p_{\textrm{T}} (\ell \ell )$ distributions are well described by mg5_amc, which relies on NLO matrix elements for 0, 1 and 2 partons in the final state. Nevertheless, mg5_amc predicts cross sections larger than those observed for the highest $p_{\textrm{T}} (\ell \ell )$ values measured in the mass ranges $106< m_{\ell \ell } <170\,\text {Ge\hspace{-.08em}V}$  for both the inclusive and 1 jet cases. Since the theoretical uncertainty is dominant in that region, a better agreement might be found using higher-order (e.g., NNLO) multiparton predictions.

The $\textsc {MiNNLO}_\textrm{PS}$ prediction provides the best global description of the data among the predictions presented in this paper. This approach, based on NNLO matrix element and pythia 8 parton shower and MPI, describes well the large $p_{\textrm{T}} (\ell \ell )$ cross sections (Fig. 6) and ratios (Fig. 17), except above 400$\,\text {Ge\hspace{-.08em}V}$, for $m_{\ell \ell }$ around the Z boson peak. The medium and low $p_{\textrm{T}} (\ell \ell )$ cross sections are also well described by $\textsc {MiNNLO}_\textrm{PS}$ which relies on parton showers, a harder primordial $k_{\textrm{T}}$ and Sudakov form factors. The same observation can be made in the one jet case. The inclusion of an NNLO matrix element reduces significantly the scale uncertainties, in particular for the inclusive cross section in for the medium $p_{\textrm{T}} (\ell \ell )$ values where the PDF uncertainty becomes significant with respect to other model uncertainties. It has to be noted that no parton shower tune uncertainty is assigned in the case of $\textsc {MiNNLO}_\textrm{PS}$ as well as in the case of mg5_amc.

We see that the Cascade predictions (mg5_amc + PB(Cascade)) involving TMDs produce a better description in the low-$p_{\textrm{T}} (\ell \ell )$ part than mg5_amc + pythia 8, which is valid for all $m_{\ell \ell }$ bins. The predicted cross section for medium $p_{\textrm{T}} (\ell \ell )$ values is 5 to 10% too low (Fig. 7). What is remarkable is that this prediction is based on TMDs obtained from totally independent data, from a fit to electron-proton deep inelastic scattering measurements performed at HERA. The high $p_{\textrm{T}} (\ell \ell )$ part is not described by the Z +0,1 jet matrix element calculations from mg5_amc with Cascade due to missing higher fixed-order calculations. The range of $p_{\textrm{T}} (\ell \ell )$ values well described extends with increasing $m_{\ell \ell }$. For the one jet case (Fig. 14), the low-$p_{\textrm{T}} (\ell \ell )$ part is mainly dominated by $\text {Z} + 2$ jet events, and the Cascade predictions are missing the contributions from the double parton scattering. It thus fails to describe the low $p_{\textrm{T}} (\ell \ell )$ region. In the low-$p_{\textrm{T}} (\ell \ell )$ region of the 1 jet case double parton scattering contributions play a significant role and thus Cascade without it cannot describe this region. The Cascade predictions give an overall good description of the ratio measurements (Fig. 17). Recently the predictions have been extended by including multi-jet merging [89] for an improved description of the full $p_{\textrm{T}} (\ell \ell )$ spectrum, shown in the Appendix A.

Within its range of validity, $p_{\textrm{T}} (\ell \ell ) < 0.2 \ m_{\ell \ell }$, the arTeMiDe prediction describes the measurements very well. For all $m_{\ell \ell }$, the low-$p_{\textrm{T}} (\ell \ell )$ distributions predicted by arTeMiDe, based on TMDs corresponding to an $\hbox {N}^3$LL approximation, are in very good agreement with the data, except for the highest masses. Figure 7 shows the prediction with and without QED FSR corrections. This underlines the importance of migrations from the Z boson peak towards lower masses, inducing the peak structure in the $p_{\textrm{T}} (\ell \ell )$ ratio distribution of Fig. 9. The remarkable agreement of the arTeMiDe prediction with the measurement at the Z boson peak is expected since the prediction relies on TMDs fitted on previous DY measurements at the Z boson peak though at lower centre of mass energies. The excellent agreement for higher $m_{\ell \ell }$ confirms the validity of the approach and in particular of the TMD factorization when the mass scale largely dominates over the transverse momentum. No prediction is provided by arTeMiDe for the 1 jet case nor for the $\varphi ^{*}_{\eta }$ cross section dependence.

Comparisons of the inclusive cross section as a function of $p_{\textrm{T}} (\ell \ell )$ with two predictions of Geneva are presented in Fig. 8 for the inclusive cross sections and in Fig. 15 for the one jet cross sections. The original prediction combining NNLL resummation on the 0-jettiness variable $\tau _0$ (Geneva-$\tau$) and NNLO corrections does not describe the data well for $p_{\textrm{T}} (\ell \ell )$ values below 40$\,\text {Ge\hspace{-.08em}V}$. This too hard $p_{\textrm{T}} (\ell \ell )$ spectrum might be related to the choice of $\alpha _\textrm{S}$, as discussed in Ref. [94]. For the high $p_{\textrm{T}} (\ell \ell )$ region, which is dominated by the fixed-order effects, the inclusion of NNLO corrections provides a good description of the measured cross section. The more recent Geneva prediction (Geneva-$q_\textrm{T}$), using a $q_\textrm{T}$ resummation at $\hbox {N}^3$LL, provides a much better description of the measured inclusive cross sections, describing very well the data in the full $p_{\textrm{T}} (\ell \ell )$ range except for middle $p_{\textrm{T}} (\ell \ell )$ values in the lowest mass bin. Here, as in $\textsc {MiNNLO}_\textrm{PS}$ case, the inclusion of NNLO corrections provides a significant reduction of the scale uncertainties, leading to very small theory uncertainties in the middle $p_{\textrm{T}} (\ell \ell )$ range. The two Geneva predictions compared with the measured one jet cross sections are similar because both use 1- jettiness in this part of the phase space. This could explain that Geneva predicts a too hard $p_{\textrm{T}} (\ell \ell )$ spectrum, similarly to the 0-jettiness inclusive case.

### $\varphi ^{*}_{\eta }$ results

**Fig. 19:**
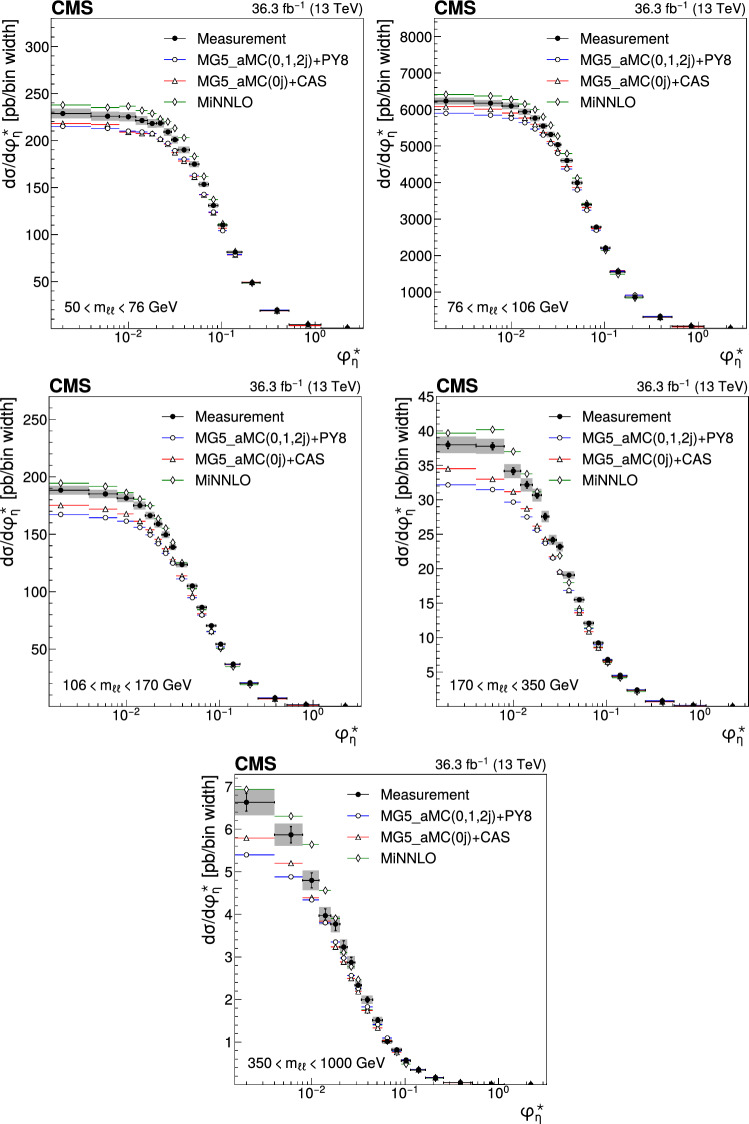
Differential cross sections in $\varphi ^{*}_{\eta } (\ell \ell )$ in various invariant mass ranges: $50< m_{\ell \ell } <76\,\text {Ge\hspace{-.08em}V}$ (upper left), $76< m_{\ell \ell } <106\,\text {Ge\hspace{-.08em}V}$ (upper right), $106< m_{\ell \ell } <170\,\text {Ge\hspace{-.08em}V}$ (middle left), $170< m_{\ell \ell } <350\,\text {Ge\hspace{-.08em}V}$ (middle right), and $350< m_{\ell \ell } <1000\,\text {Ge\hspace{-.08em}V}$ (lower). The measurement is compared with mg5_amc (0, 1, and 2 jets at NLO) + pythia 8 (blue dots), $\textsc {MiNNLO}_\textrm{PS}$ (green diamonds) and mg5_amc (0 jet at NLO)+ PB (Cascade) (red triangles). Details on the presentation of the results are given in Fig. 5 caption

**Fig. 20:**
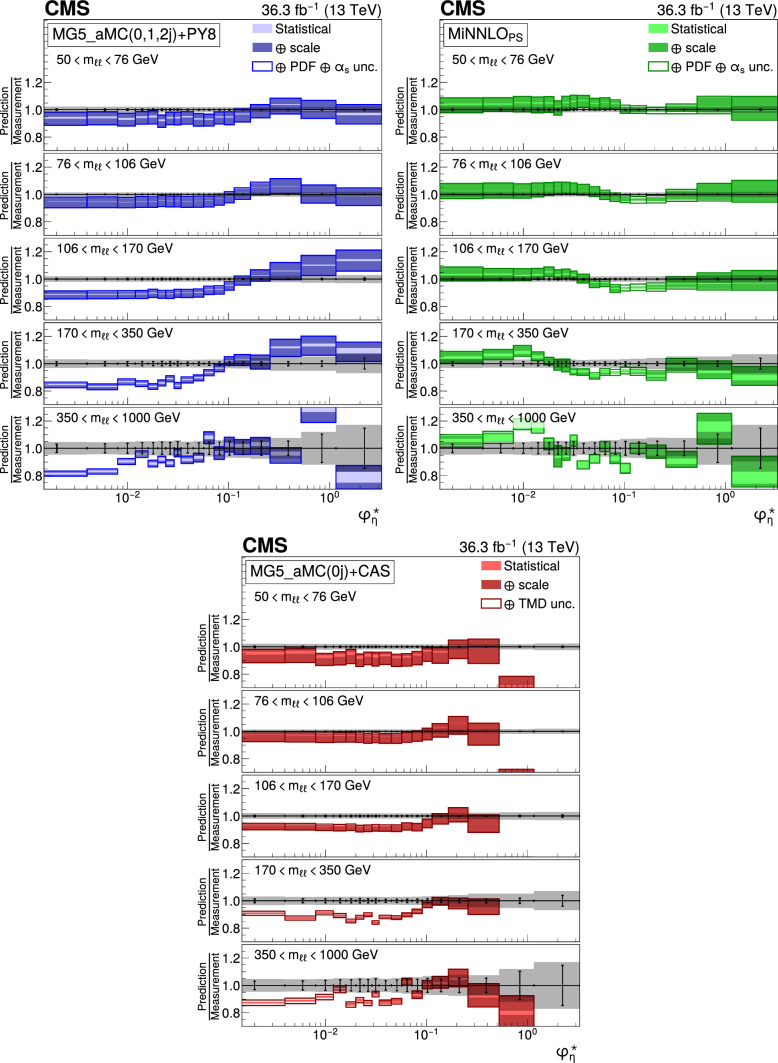
Comparison of the differential cross sections in $\varphi ^{*}_{\eta } (\ell \ell )$ to predictions in various $m_{\ell \ell }$ ranges. The measurement is compared with mg5_amc (0, 1, and 2 jets at NLO) + pythia 8 (upper left), $\textsc {MiNNLO}_\textrm{PS}$ (upper right) and mg5_amc + PB (Cascade) (lower). Details on the presentation of the results are given in Fig. 6 caption

**Fig. 21:**
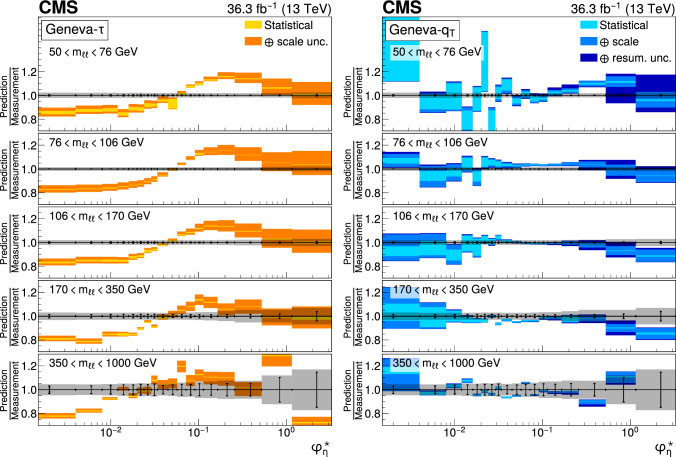
Comparison of the differential cross sections in $\varphi ^{*}_{\eta } (\ell \ell )$ to predictions in various $m_{\ell \ell }$ ranges. The measurement is compared with Geneva-$\tau$ (left) and Geneva-$q_\textrm{T}$ (right) predictions. Details on the presentation of the results are given in Fig. 8 caption

**Fig. 22:**
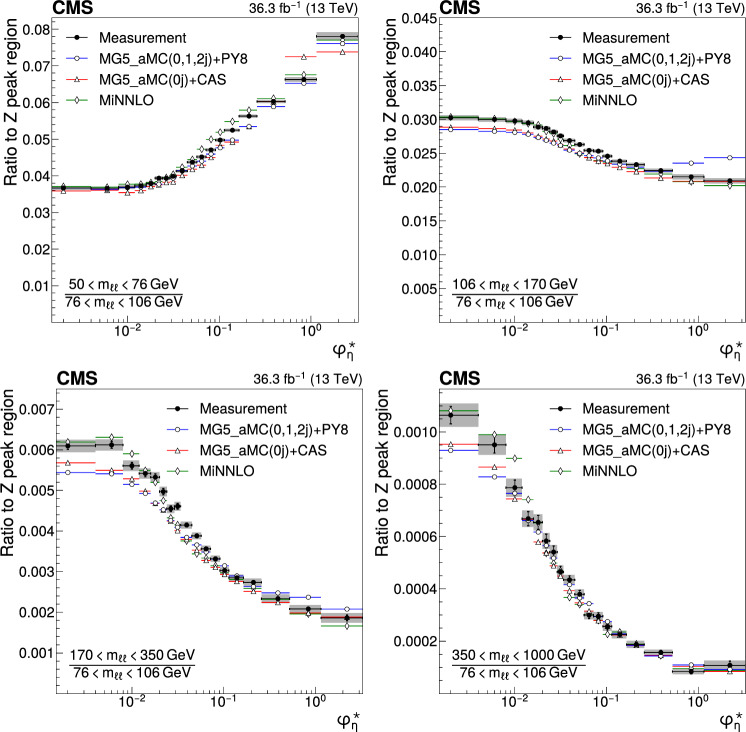
Ratios of differential cross sections in $\varphi ^{*}_{\eta } (\ell \ell )$ for invariant mass ranges with respect to the peak region $76< m_{\ell \ell } <106\,\text {Ge\hspace{-.08em}V}$: $50< m_{\ell \ell } <76\,\text {Ge\hspace{-.08em}V}$ (upper left), $106< m_{\ell \ell } <170\,\text {Ge\hspace{-.08em}V}$ (upper right), $170< m_{\ell \ell } <350\,\text {Ge\hspace{-.08em}V}$ (lower left), and $350< m_{\ell \ell } <1000\,\text {Ge\hspace{-.08em}V}$ (lower right). The measurement is compared with mg5_amc (0, 1, and 2 jets at NLO) + pythia 8 (blue dots), $\textsc {MiNNLO}_\textrm{PS}$ (green diamonds) and mg5_amc (0 jet at NLO)+ PB (Cascade) (red triangles). Details on the presentation of the results are given in Fig. 5 caption

**Fig. 23:**
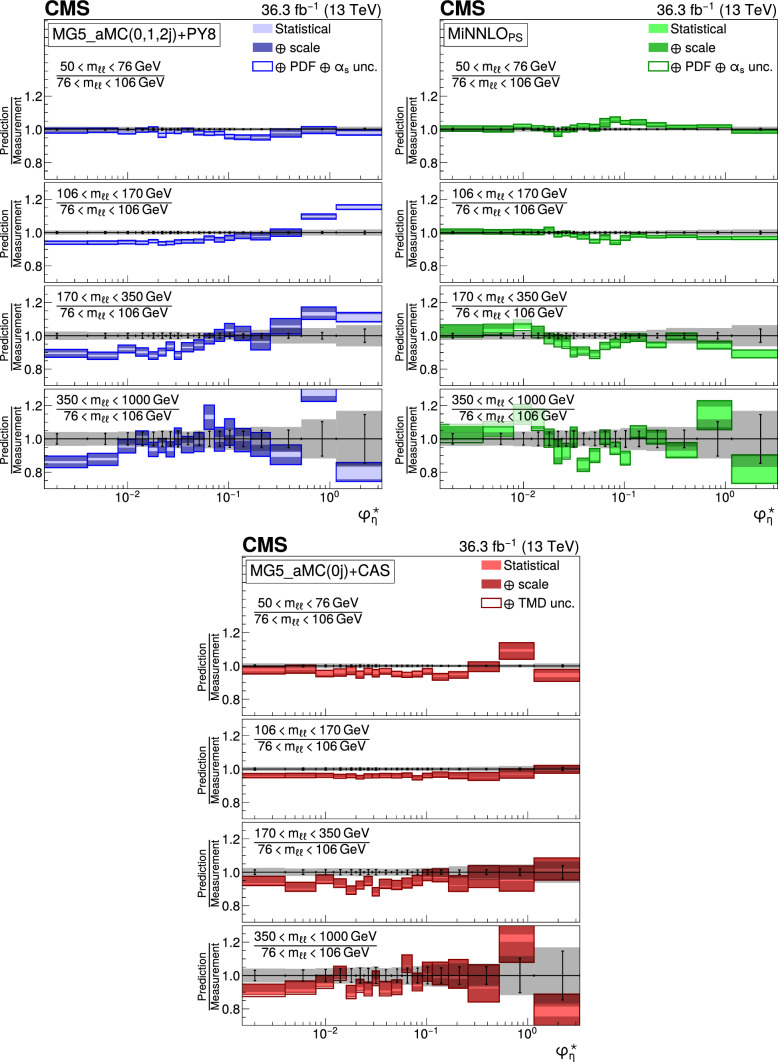
Ratios of differential cross sections in $\varphi ^{*}_{\eta } (\ell \ell )$ for invariant $m_{\ell \ell }$ with respect to the peak region $76< m_{\ell \ell } <106\,\text {Ge\hspace{-.08em}V}$. Compared to model predictions from mg5_amc (0, 1, and 2 jets at NLO) + pythia 8 (upper left), $\textsc {MiNNLO}_\textrm{PS}$ (upper right) and mg5_amc (0 jet at NLO) + PB (Cascade) (lower). Details on the presentation of the results are given in Fig. 6 caption

**Fig. 24:**
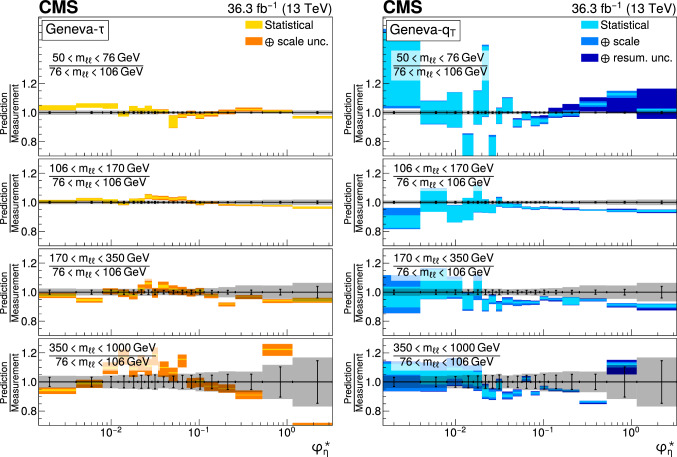
Ratios of differential cross sections in $\varphi ^{*}_{\eta } (\ell \ell )$ for invariant $m_{\ell \ell }$ with respect to the peak region $76< m_{\ell \ell } <106\,\text {Ge\hspace{-.08em}V}$. Compared to model predictions from Geneva-$\tau$ (left) and Geneva-$q_\textrm{T}$ (right). Details on the presentation of the results are given in Fig. 8 caption

The $\varphi ^{*}_{\eta }$ variable is highly correlated with $p_{\textrm{T}} (\ell \ell )$ and it offers a complementary access to the underlying QCD dynamics. Being based only on angle measurements of the final-state charged leptons, the $\varphi ^{*}_{\eta }$ variable can be measured with greater accuracy which allows us to include the muon channel for all $m_{\ell \ell }$ ranges. Figure 19 presents the inclusive differential cross sections in $\varphi ^{*}_{\eta }$ for the same invariant mass ranges as above and comparisons to models. More complete comparisons to model predictions are presented as ratios of the prediction divided by the measurement in Figs. 20 and 21. The results are discussed below. The ratio of these differential cross sections for various $m_{\ell \ell }$ ranges are computed with respect to the same distribution in the Z peak region. They are shown in Fig. 22 and further compared with models in Figs. 23 and 24.

The $\varphi ^{*}_{\eta }$ distributions are monotonic functions, in particular they do not present a peak structure as measured in the $p_{\textrm{T}} (\ell \ell )$ distributions. At small values, the $\varphi ^{*}_{\eta }$ distributions contain a plateau whose length decreases with increasing $m_{\ell \ell }$, and more generally the $\varphi ^{*}_{\eta }$ distributions fall more rapidly with increasing $m_{\ell \ell }$ as clearly shown in Fig. 19. Because the lepton direction is much less affected by QED FSR than the energy, the effect of migrations from the Z boson mass bin towards lower masses is relatively invisible in the $\varphi ^{*}_{\eta }$ shape as highlighted by the ratio distribution in Fig. 22 (upper left).

Since $\varphi ^{*}_{\eta }$ is highly correlated with $p_{\textrm{T}} (\ell \ell )$, the comparison of the $\varphi ^{*}_{\eta }$ distributions to theoretical predictions leads to the same basic observations and remarks as related above. The mg5_amc + pythia 8 prediction describes the measured $\varphi ^{*}_{\eta }$ distributions well globally and predicts a too small cross section in the region sensitive to gluon resummation, i.e., $\varphi ^{*}_{\eta } \lesssim 0.1$ on the Z boson mass peak, as shown in Fig. 20. The increase of this disagreement for higher $m_{\ell \ell }$ is also observed, clearly visible in the ratio distributions of Fig. 23.

As for the $p_{\textrm{T}} (\ell \ell )$ distributions, the $\textsc {MiNNLO}_\textrm{PS}$ prediction provides the best global description of the data (Fig. 20). In contrast to the disagreement for $p_{\textrm{T}} (\ell \ell )$ above 400$\,\text {Ge\hspace{-.08em}V}$ for $m_{\ell \ell }$ around the Z peak that appeared both in the inclusive case (Fig. 6) and in the one jet case (Fig. 17) the large $\varphi ^{*}_{\eta }$ values are well described by $\textsc {MiNNLO}_\textrm{PS}$. The inclusion of NNLO corrections reduces scale uncertainties making the PDF uncertainty dominant for medium $\varphi ^{*}_{\eta }$ values in the central $m_{\ell \ell }$ bins. The PDF uncertainty is significantly reduced in the ratio distributions (Fig. 23) leading to remarkable prediction precision of the level of 1.5% in several bins.

The mg5_amc + PB(Cascade) prediction describes well the measured shapes for $\varphi ^{*}_{\eta } \lesssim 0.1$ in all $m_{\ell \ell }$ bins (Fig. 20). This contrasts with the description of the $p_{\textrm{T}} (\ell \ell )$ dependence by the same prediction (Fig. 7), owing to the washing out of the details of the $p_{\textrm{T}} (\ell \ell )$ distribution in the $\varphi ^{*}_{\eta }$ distribution. The normalisation of the prediction is good for the Z boson mass peak region but underestimates more and more the cross section with increasing $m_{\ell \ell }$, in a way relatively close to mg5_amc predictions. The ratio distributions (Fig. 23) also illustrate this, but a compensation effect leads to predictions in agreement over the full $\varphi ^{*}_{\eta }$ range.

The measured cross sections as a function of $\varphi ^{*}_{\eta }$ are compared with Geneva predictions in Fig. 21. Similar to previous discussions of the $p_{\textrm{T}} (\ell \ell )$ distributions, Geneva-$q_\textrm{T}$ improves significantly the description of the data with respect to Geneva-$\tau$. The discrepancy of Geneva-$q_\textrm{T}$ for low $p_{\textrm{T}} (\ell \ell )$ values in the two lowest $m_{\ell \ell }$ bins is smoothed here leading to a global agreement everywhere. The cross section ratio distributions of the different $m_{\ell \ell }$ bins over the Z boson mass peak bin, as a function of $\varphi ^{*}_{\eta }$ are shown in Fig. 24. Here both Geneva predictions provide a good description of the measurements. This indicates that, although the precise shape in $\varphi ^{*}_{\eta }$ is not well reproduced by Geneva-$\tau$, the scale dependence is well described over the large range covered by the present measurement.

The differential cross section measurements are presented in the HEPData entry [100].

## Summary

Measurements of differential Drell–Yan cross sections in proton–proton collisions at $\sqrt{s} = 13\,\text {Te\hspace{-.08em}V}$ in the dielectron and dimuon final states are presented, using data collected with the CMS detector, corresponding to an integrated luminosity of 36.3$\,\text {fb}^{-1}$. The measurements are corrected for detector effects and the two leptonic channels are combined. Differential cross sections in the dilepton transverse momentum, $p_{\textrm{T}} (\ell \ell )$, and in the lepton angular variable $\varphi ^{*}_{\eta }$ are measured for different values of the dilepton mass, $m_{\ell \ell }$, between 50$\,\text {Ge\hspace{-.08em}V}$ and 1$\,\text {Te\hspace{-.08em}V}$. To highlight the evolution with the dilepton mass scale, ratios of these distributions for various masses are presented. In addition, dilepton transverse momentum distributions are shown in the presence of at least one jet within the detector acceptance.

The rising behaviour of the Drell–Yan inclusive cross section at small $p_{\textrm{T}} (\ell \ell )$ is attributed to soft QCD radiations, whereas the tail at large $p_{\textrm{T}} (\ell \ell )$ is only expected to be well described by models relying on higher-order matrix element calculations. Therefore, this variable provides a good sensitivity to initial-state QCD radiations and can be compared with different predictions relying on matrix element calculations at different orders and using different methods to resum the initial-state soft QCD radiations. The measurements show that the peak in the $p_{\textrm{T}} (\ell \ell )$ distribution, located around 5$\,\text {Ge\hspace{-.08em}V}$, is not significantly modified by changing the $m_{\ell \ell }$ value in the covered range. However, for higher values of $m_{\ell \ell }$ above the peak, the $p_{\textrm{T}} (\ell \ell )$ distributions fall less steeply.

The $\varphi ^{*}_{\eta }$ variable, highly correlated with $p_{\textrm{T}} (\ell \ell )$, offers a complementary access to the underlying QCD dynamics. Since it is based only on angle measurements of the final-state charged leptons, it offers, a priori, measurements with greater accuracy. However, these measurements demonstrate that the $\varphi ^{*}_{\eta }$ distributions discriminate between the models less than the $p_{\textrm{T}} (\ell \ell )$ distributions, since they wash out the peak structure of the $p_{\textrm{T}} (\ell \ell )$ distributions, which reflect the initial-state QCD radiation effects in a more detailed way.

This publication presents comparisons of the measurements to six predictions using different treatments of soft initial-state QCD radiations. Two of them, mg5_amc + pythia 8 and $\textsc {MiNNLO}_\textrm{PS}$, are based on a matrix element calculation merged with parton showers. Two others, arTeMiDe and Cascade use transverse momentum dependent parton distributions (TMD). Finally, Geneva combines a higher-order resummation with a Drell–Yan calculation at next-to-next-to-leading order (NNLO), in two different ways. One carries out the resummation at next-to-next-to-leading logarithm in the 0-jettiness variable $\tau _0$, the other at $\mathrm {N^3LL}$ in the $q_\textrm{T}$ variable.

The comparison of the measurement with the mg5_amc + pythia 8 Monte Carlo predictions using matrix element calculations including $\text {Z} + 0,1,2$ partons at next-to-leading order (NLO) merged with a parton shower, shows generally good agreement, except at $p_{\textrm{T}} (\ell \ell )$ values below 10$\,\text {Ge\hspace{-.08em}V}$ both for the inclusive and one jet cross sections. This disagreement is enhanced for masses away from the Z mass peak and is more pronounced for the higher dilepton masses, reaching 20% for the highest mass bin.

The $\textsc {MiNNLO}_\textrm{PS}$ prediction provides the best global description of the data among the predictions presented in this paper, both for the inclusive and the one jet cross sections. This approach, based on NNLO matrix element and pythia 8 parton shower and MPI, describes well the large $p_{\textrm{T}} (\ell \ell )$ cross sections and ratios, except for $p_{\textrm{T}} (\ell \ell )$ values above 400$\,\text {Ge\hspace{-.08em}V}$ for dilepton masses around the Z mass peak. A good description of the medium and low $p_{\textrm{T}} (\ell \ell )$ cross sections is obtained using a modified primordial $k_{\textrm{T}}$ parameter of the CP5 parton shower tune.

mg5_amc + PB(Cascade) predictions are based on Parton Branching TMDs obtained only from a fit to electron-proton deep inelastic scattering measurements performed at HERA. These TMDs are merged with NLO matrix element calculations. Low $p_{\textrm{T}} (\ell \ell )$ values are globally well described but with too low cross sections at medium $p_{\textrm{T}} (\ell \ell )$ values. This discrepancy increases with increasing $m_{\ell \ell }$ in a way similar to the mg5_amc + pythia 8 predictions. The high part of the $p_{\textrm{T}} (\ell \ell )$ distribution is not described by Cascade due to missing higher fixed-order terms. The model can not describe the low $p_{\textrm{T}} (\ell \ell )$ region of the cross section in the presence of one jet due to the missing double parton scattering contributions. The recent inclusion of multi-jet merging allows a larger $p_{\textrm{T}} (\ell \ell )$ region to be described as well.

arTeMiDe provides predictions based on TMDs extracted from previous measurements including the Drell-Yan transverse momentum cross section at the LHC at the Z mass peak. By construction, the validity of arTeMiDe predictions are limited to the range $p_{\textrm{T}} (\ell \ell ) < 0.2 \ m_{\ell \ell }$. In that range, they describe well the present measurements up to the highest dilepton masses.

The Geneva prediction, combining resummation in the 0-jettiness variable $\tau _0$ (Geneva-$\tau$) and NNLO matrix element does not describe the measurement well for $p_{\textrm{T}} (\ell \ell )$ values below 40$\,\text {Ge\hspace{-.08em}V}$. For the high $p_{\textrm{T}} (\ell \ell )$ region the inclusion of NNLO in the matrix element provides a good description of the measured cross section. The recent Geneva prediction (Geneva-$q_\textrm{T}$), using a $q_\textrm{T}$ resummation, provides a much better description of the measured inclusive cross sections, describing very well the data in the full $p_{\textrm{T}} (\ell \ell )$ range except for middle $p_{\textrm{T}} (\ell \ell )$ values in the lowest mass bin. Both Geneva approaches predict too hard $p_{\textrm{T}} (\ell \ell )$ spectra for the one jet cross sections.

The ratio distributions presented in this paper confirm most of the observations based on the comparison between the measurement and the predictions at the cross section level. The observed scale dependence is well described by the different models. Furthermore the partial cancellation of the uncertainties in the cross section ratios allows a higher level of precision to be reached for both the measurement and the predictions.

The present analysis shows the relevance of measuring the Drell–Yan cross section in a wide range in dilepton masses to probe the interplay between the transverse momentum and the mass scales of the process. Important theoretical efforts have been made during the last decade to improve the detailed description of high energy processes involving multiple scales and partonic final states. The understanding of the Drell–Yan process directly benefited from these developments. The present paper shows that they individually describe the measurements well in the regions they were designed for. Nevertheless, no model is able to reproduce all dependencies over the complete covered range. Further progress might come from combining these approaches.

## Figures and Tables

**Fig. 25:**
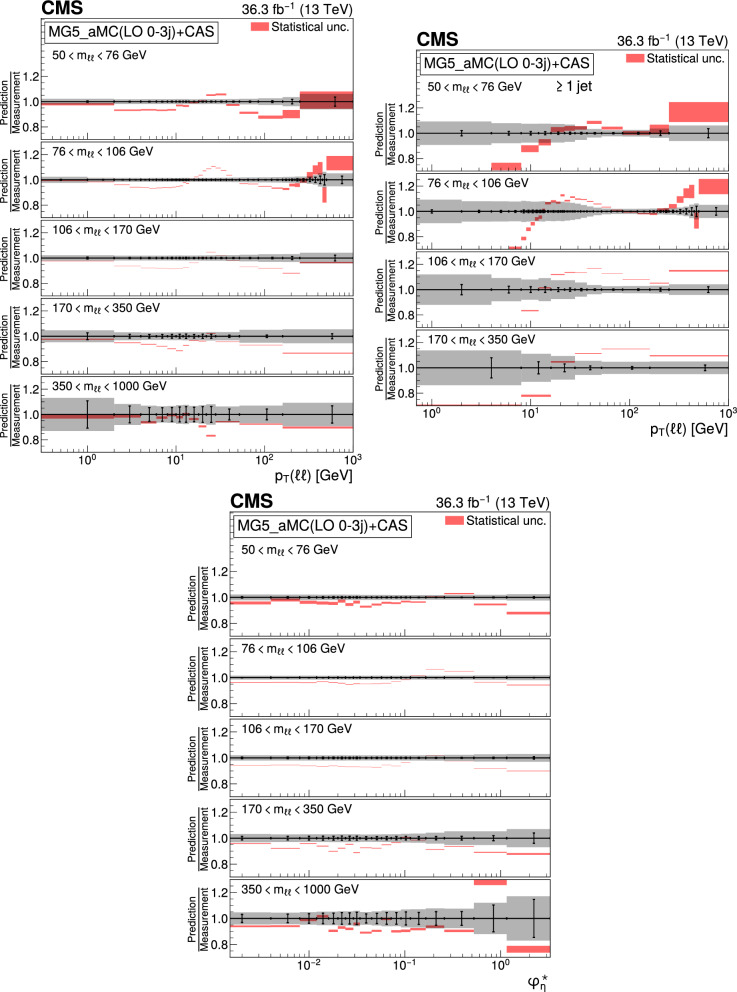
The ratio of mg5_amc (0, 1, 2 and 3 jets at LO) + PB (Cascade) predictions to the measured differential cross sections in $p_{\textrm{T}} (\ell \ell )$ (upper left), in $p_{\textrm{T}} (\ell \ell )$ for the one or more jets case (upper right), and in $\varphi ^{*}_{\eta }$ (bottom) are presented for various $m_{\ell \ell }$ ranges. The error bars correspond to the statistical uncertainty of the measurement and the shaded bands to the total experimental uncertainty. The light color band around Cascade prediction corresponds to the statistical uncertainty of the simulation

**Fig. 26:**
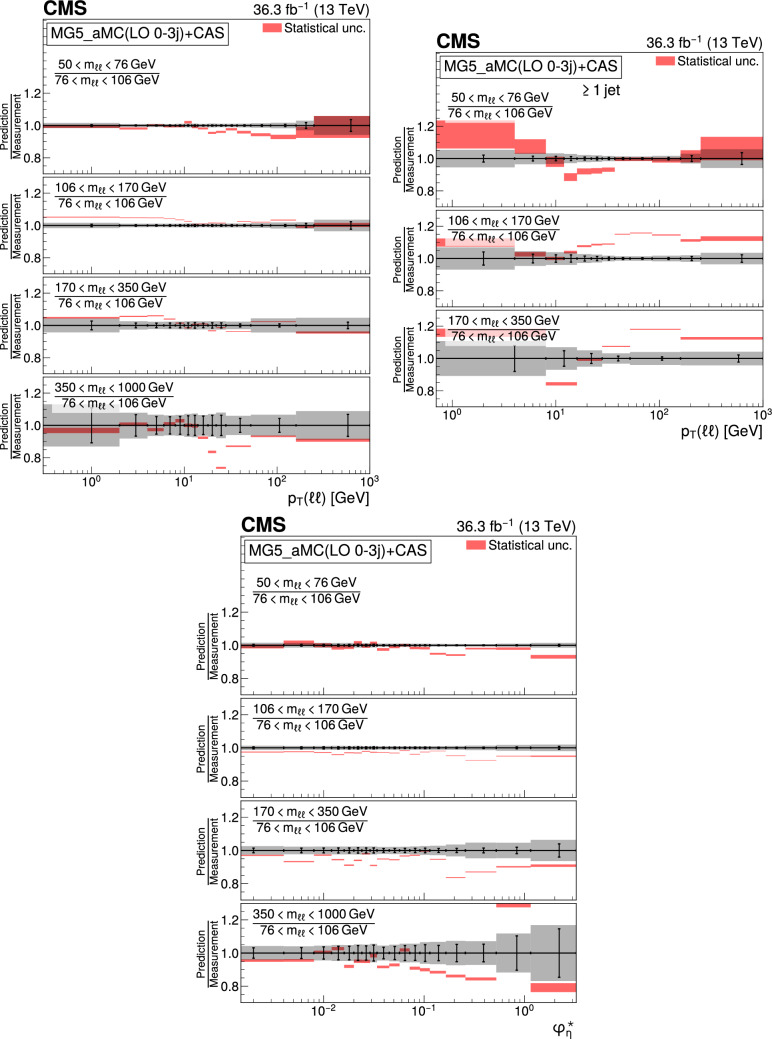
The ratio of mg5_amc (0, 1, 2 and 3 jets at LO) + PB (Cascade) predictions to the ratios of differential cross sections for $m_{\ell \ell }$ ranges with respect to the peak region in $p_{\textrm{T}} (\ell \ell )$ (upper left), in $p_{\textrm{T}} (\ell \ell )$ for the one or more jets case (upper right), and in $\varphi ^{*}_{\eta }$ (bottom). The error bars correspond to the statistical uncertainty of the measurement and the shaded bands to the total experimental uncertainty. The light color band around Cascade prediction corresponds to the statistical uncertainty of the simulation

## Data Availability

This manuscript has no associated data or the data will not be deposited. [Authors’ comment: Release and preservation of data used by the CMS Collaboration as the basis for publications is guided by the CMS policy as stated in https://cms-docdb.cern.ch/cgi-bin/PublicDocDB/RetrieveFile?docid=6032 &filename=CMSDataPolicyV1.2.pdf &version=2 CMS data preservation, re-use and open access policy.]

## Appendix A: Comparisons to other models

In this section, comparisons of the obtained measurement results with predictions from a more recent parton branching (PB) TMD method from Cascade are presented. The predictions are based on mg5_amc ME up to three partons at LO in QCD with multi-jet merging [89]. The ratio of the predictions over the data are presented in Fig. 25. The comparisons to predictions for the ratio of the cross sections for invariant masses outside the Z boson peak to the distribution within the Z boson peak ($76< m_{\ell \ell } <106\,\text {Ge\hspace{-.08em}V}$) are shown in Fig. 26.
